# The Basis for Weekly Insulin Therapy: Evolving Evidence With Insulin Icodec and Insulin Efsitora Alfa

**DOI:** 10.1210/endrev/bnad037

**Published:** 2024-01-16

**Authors:** Julio Rosenstock, Rattan Juneja, John M Beals, Julie S Moyers, Liza Ilag, Rory J McCrimmon

**Affiliations:** Velocity Clinical Research at Medical City, Dallas, TX 75230, USA; Lilly Diabetes and Obesity, Eli Lilly and Company, Indianapolis, IN 46225, USA; Lilly Diabetes and Obesity, Eli Lilly and Company, Indianapolis, IN 46225, USA; Lilly Diabetes and Obesity, Eli Lilly and Company, Indianapolis, IN 46225, USA; Lilly Diabetes and Obesity, Eli Lilly and Company, Indianapolis, IN 46225, USA; School of Medicine, University of Dundee, Dundee DD1 9SY, Scotland, UK

**Keywords:** basal insulin, once-weekly insulin, insulin icodec, insulin efsitora, peak-to-trough ratio, hypoglycemia

## Abstract

Basal insulin continues to be a vital part of therapy for many people with diabetes. First attempts to prolong the duration of insulin formulations were through the development of suspensions that required homogenization prior to injection. These insulins, which required once- or twice-daily injections, introduced wide variations in insulin exposure contributing to unpredictable effects on glycemia. Advances over the last 2 decades have resulted in long-acting, soluble basal insulin analogues with prolonged and less variable pharmacokinetic exposure, improving their efficacy and safety, notably by reducing nocturnal hypoglycemia. However, adherence and persistence with once-daily basal insulin treatment remains low for many reasons including hypoglycemia concerns and treatment burden. A soluble basal insulin with a longer and flatter exposure profile could reduce pharmacodynamic variability, potentially reducing hypoglycemia, have similar efficacy to once-daily basal insulins, simplify dosing regimens, and improve treatment adherence. Insulin icodec (Novo Nordisk) and insulin efsitora alfa (basal insulin Fc [BIF], Eli Lilly and Company) are 2 such insulins designed for once-weekly administration, which have the potential to provide a further advance in basal insulin replacement. Icodec and efsitora phase 2 clinical trials, as well as data from the phase 3 icodec program indicate that once-weekly insulins provide comparable glycemic control to once-daily analogues, with a similar risk of hypoglycemia. This manuscript details the technology used in the development of once-weekly basal insulins. It highlights the clinical rationale and potential benefits of these weekly insulins while also discussing the limitations and challenges these molecules could pose in clinical practice.

## Graphical Abstract

**Graphical Abstract:**
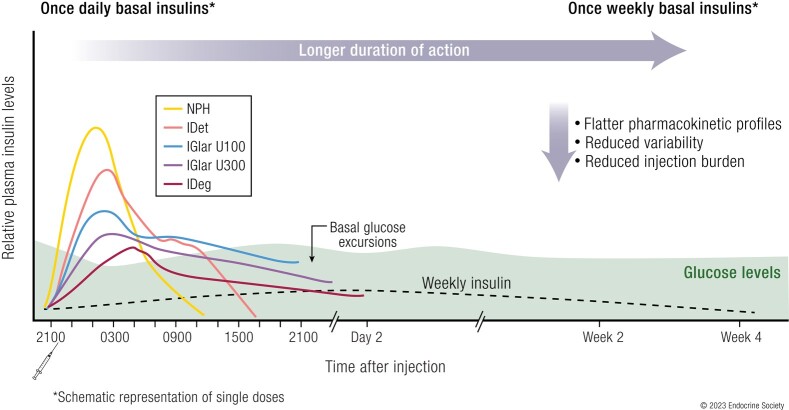

**Essential Points**

- Over the last 25 years, long-acting soluble once-daily basal insulin analogues have improved the efficacy and safety of treatment compared to earlier insulin formulations
- Despite availability of once-daily insulins, adherence and persistence on therapy are lower than desired
- A once-weekly insulin with a flat pharmacokinetic profile could reduce the injection burden and glycemic variability, which may translate to better adherence and persistence to insulin treatment
- Insulin icodec, an acylated insulin analogue, and insulin efsitora alfa, an Fc-fused insulin receptor agonist, are in late-stage clinical development as once-weekly insulins
- Available clinical data from insulin icodec and insulin efsitora show comparable glycemic control to once-daily analogues with a generally similar risk of hypoglycemia
- Education around the new dosing regimens for once-weekly insulins will be needed to facilitate safe and effective use of these molecules

Over the past century, since the discovery of insulin, tremendous advances have been made to create insulin molecules that can more closely match physiologic insulin secretion profiles. Therapies have evolved from the initial crude pancreatic extracts with beef or pork sources to biosynthetic molecules allowing for amino acid changes and chemical modification. The quest has been, and continues to be, the achievement of exogenous insulins that mimic endogenous secretion of both bolus and basal time-action.

The first attempts to prolong the duration of time-action focused on altering the formulations of short-acting insulin. Beginning in the 1930s, intermediate- to long-acting formulations were developed by the addition of excess zinc (lente and ultralente) and/or protamine (neutral protamine Hagedorn [NPH] insulin and protamine zinc insulin) (1). These amorphous and/or crystalline formulations were designed to slow the absorption of insulin from the subcutaneous (SC) depot but required the patient to resuspend prior to twice-daily or once-daily administration. These formulations were associated with unpredictable glucose control and higher than desired rates of hypoglycemia, which were attributed to (a) insufficient time-action that required multiple daily injections; (b) challenges calculating dose requirements due to high glucose control variability; (c) higher than desired variability in dissolution of the suspension in the heterogeneous SC space; and (d) inconsistent homogenization of the suspension (2). These limitations were magnified further in a basal/bolus dosing regimen.

Over the last 25 years, major advances in insulin engineering, coupled with creative formulation designs that enhanced insulin self-association properties, have resulted in the development of long-acting, soluble basal insulin therapies that prolong pharmacokinetic (PK) exposure, flatten the insulin exposure profile, and lessen variability over a 24-hour period to help mimic endogenous insulin action. These strategies produced the first generation of basal insulin analogues, insulin glargine (IGlar U100; Sanofi S/A) and insulin detemir (IDet U100; Novo Nordisk A/S). These molecules, while seen as an advance as compared to NPH in time-action (IGlar) or variability (IDet), did not achieve all the properties desired of a basal insulin (1). IGlar U100 offered a significant development in basal insulin therapy by demonstrating a solution formulation with reduced nocturnal hypoglycemia compared to NPH due to its flatter metabolic activity profile and by introducing the concept of a simple treat-to-target dosing regimen for weekly titration based on daily fasting glucose levels that changed the standards of care for insulin management in type 2 diabetes (T2D) (3-5). However, despite these advances, some patients still require twice-daily dosing of IGlar U100 or IDet (1). Subsequent iterations of these first-generation basal insulin analogues, by formulation and/or chemical modifications, yielded a longer acting second generation of analogues: IGlar U300 (Sanofi) and insulin degludec (IDeg U100, IDeg U200; Novo Nordisk). These second-generation basal insulins deliver a true once-daily basal profile for nearly all patients.

Despite the availability of these improved once-daily options, effective basal insulin therapy can be challenging due to dose administration timing, frequent dose adjustments, and a high number of injections (∼365/year) (1). Collectively, these affect adherence and result in only approximately 5% to 45% of patients achieving desired glycemic goals in the real world (6-8).

In the past decade, protein engineers have harvested learnings from these first- and second-generation basal insulin analogues, as well as other clinically tested basal insulins (eg, insulin peglispro; Eli Lilly and Company), to design molecules that integrate novel strategies to create a third generation of basal insulin therapies. The goal of these third-generation insulins is to extend the time-action profile to allow for once-weekly administration and to more closely mimic endogenous basal insulin distribution profiles. The development of a once-weekly basal insulin with a longer, flatter exposure profile, coupled with controlled tissue distribution properties and attenuated potency at the insulin receptor (IR) could reduce variability by controlling fluctuations in glucose levels during the week, while maintaining an acceptable and manageable hypoglycemia profile. However, an important limitation with these insulins is the inability to rapidly adapt to changes in insulin requirements, which the body achieves with controlled endogenous insulin secretion.

An additional premise to consider is that patients’ preference for fewer injections may facilitate improved insulin acceptance, adherence, and treatment persistence as patients would likely prefer 52 instead of 365 injections per year. In patients with type 1 diabetes (T1D), weekly insulins could benefit, not only with the reduction in injection number, but potentially from a reduction in the frequency of recurrent diabetes ketoacidosis in those at highest risk, for example, adolescents whose compliance with insulin therapy can be inconsistent. However, to use these insulins safely, health care providers and patients will be required to learn new dosing regimens that are unfamiliar today. These include (a) the potential need for an initial one-time loading dose, (b) the need to learn how to transition between once-daily and once-weekly insulins, (c) patient management for missed doses or accidental dosing errors, and (d) patient management during hospitalizations, surgery, fasting, and exercise.

This review is intended to provide in-depth information that describes the technology supporting the development of once-weekly basal insulins, and address the strategies explored to safely create a circulating “pool” or “reservoir” of insulin capable of engaging the IR over a weekly time frame and mimicking the effects of endogenous insulin action. It highlights the clinical rationale and potential benefits of weekly insulins while also and importantly, discusses the challenges these molecules could pose for clinical practice.

## Terminology With Exogenously Administered Insulins

**Figure 1.**
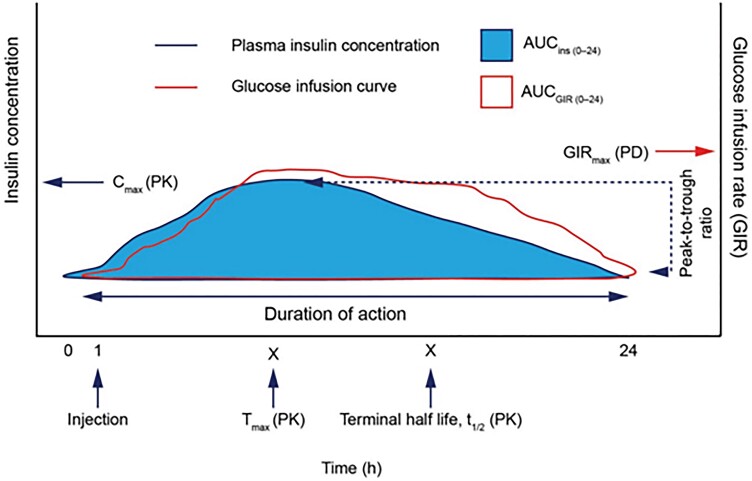
Insulin pharmacokinetics. Schematic PK/PD profile for basal insulin after administration of a single dose after maintenance phase (steady state) has been achieved highlighting key PK/PD parameters. P/T ratio: difference between the highest and lowest concentration of injected insulin at steady state; and t_1/2_: the time it takes for 50% of the drug to be eliminated relative to the C_max_. AUC, area under the curve; C_max_, peak insulin concentration reached; GIR, glucose infusion rate; GIR_max_, time of maximum glucose infusion rate; INS, insulin (analogue) concentration; PD, pharmacodynamic; PK, pharmacokinetic; T_max_, time when peak insulin concentration is reached. Reproduced with permission from Heise and Meneghini (9).

Exogenously administered basal insulins have been designed to mimic the prolonged time-action of secreted endogenous insulin, that is, create a PK profile (serum insulin concentration) for insulin that concomitantly drives intermeal pharmacodynamic (PD) effects on glucose. In this context, key terms will be used to describe therapeutic basal insulin properties (Fig. 1).

With the development of ultra long-acting basal insulins, clinicians can be concerned with insulin doses overlapping or accumulating; consequently, it is important to understand the concept of ***insulin “steady state.”*** This concept refers to a state where a dynamic equilibrium in insulin concentration exists within therapeutic limits between doses. To reach steady-state conditions with basal insulins, ***controlled accumulation*** is used, wherein, circulating insulin levels build on the remaining insulin from previous injections prior to elimination. The amount of accumulation is dependent on the half-life (t_1/2_) of the basal insulin, the insulin dose, and the frequency of dosing. With consistent dosing, a steady state is eventually achieved. Typically, a time period equivalent to 3 to 5 half-lives is required to reach steady state. Depending on the rigor of the definition, PK levels reach approximately 90% of the steady-state concentration after 3×t_1/2_ and approximately 99% after 5×t_1/2_ (9). A level of 90% (∼3×t_1/2_) is considered by many to be the threshold for clinically relevant steady state. Once at steady state, insulin levels will not increase further, as long as similar doses are administered at appropriately spaced intervals relative to the half-life of the insulin (9). Conversely, increasing insulin dose before steady state is reached could result in overinsulinization and induce hypoglycemia.

**Figure 2.**
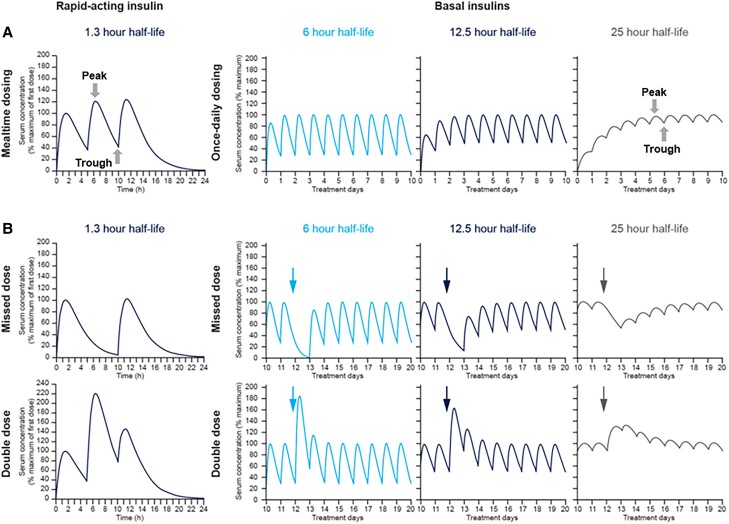
PK profiles of rapid-acting insulin and basal insulins. A, PK profile of a rapid-acting insulin analogue with a t_1/2_ of 1.3 hours (left) and basal insulins (right) with a t_1/2_ of 6 hours (NPH insulin), 12.5 hours (insulin glargine U100), or 25 hours (insulin degludec). B, Effect of missed dosing and double dosing on PK profiles of rapid-acting insulin and basal insulins at steady state. As shown in the figure, the effects of missed or double dosing are greatest with basal insulin having a shorter half-life. NPH, neutral protamine Hagedorn; PK, pharmacokinetic. Reproduced with permission from Heise and Meneghini (9).

***Peak-to-trough (P/T) ratio***  *r*efers to the difference between the peak and nadir concentrations of the injected insulin. P/T ratio is commonly used only in the context of therapeutic insulins since endogenously secreted insulin in individuals without diabetes closely and constantly matches glucose excursions. Notably, a ***high*** P/T ratio is desirable for a given dose of rapid-acting, prandial insulin and a ***low*** P/T ratio is desirable for a basal insulin (see Fig. 1 and  2A). One of the consequences of prolonging insulin time-action is enabling therapeutic accumulation and the subsequent flattening of the PK profile, resulting in a lower P/T ratio (9). The P/T ratio reflects variability, which is affected by the rate of absorption, the molecule's half-life, and the dosing interval of the insulin. A low P/T ratio indicates the insulin has a consistent plasma exposure profile and thus, a more predictable concentration of insulin available between dosing intervals (9). If appropriately generated, a flatter PK profile can decrease within- and between-day glucose variability, potentially reducing the risk of hypoglycemia, as well as enhancing patient satisfaction because the PD effect will be more predictable.

With current basal insulins, this attribute was manifested in the second-generation once-daily basal insulin analogues, IDeg and IGlar U300, which demonstrated longer time-action profiles and lower hypoglycemia risk compared to IGlar U100 (10-16). Iterations in basal insulin have led to both a reduction in nocturnal and daytime hypoglycemia (1), resulting in diabetes treatment guidelines recommending IDeg and IGlar U300 as preferred basal insulin therapies (17).

***Loading dose/one-time starting dose*** refers to an initial one-time dose used to shorten the time to reach steady state. For an insulin with a very long half-life, such as weekly insulins, a loading dose may be useful in rapidly achieving efficacious insulin concentrations to safely enable patient-tailored insulin titrations to reach glucose targets. Loading doses are not used with current basal insulins; however, loading doses were used for beef ultralente insulin, an early long-acting basal insulin in certain circumstances to shorten time to steady state (18).

## Physiological Basis for Basal Insulin Replacement

To help understand why therapeutic once-weekly basal insulins could be an advantage in clinical practice, it is useful to identify the similarities and differences between endogenously released and SC administered therapeutic insulin with regard to signaling, distribution, clearance, and time-action. It is, however, important to note that even the best therapeutic basal insulins fail to truly mimic pancreatic-secreted insulin (19).

### Endogenous Insulin

#### Structure

Mature endogenous insulin is a 2-chain hormone, composed of 51 amino acids, that is enzymatically derived from a single-chain proinsulin in the β cell of the pancreas (2). The self-association and zinc-binding properties of mature insulin facilitate storage in the secretory granules as stable hexamers. On secretion from the pancreas into the portal vein, hexameric insulin dissociates into the active monomeric conformation (20).

#### Receptor signaling

Monomeric insulin signals through the IR, a transmembrane tyrosine kinase receptor, with an extracellular α-subunit and an intracellular β-subunit (21). Insulin binding to the α-subunits elicits a series of phosphorylation events, which have been extensively reviewed previously (21, 22). These phosphorylation events mediate pleiotropic intracellular activities including, but not limited to, induction of glycogenesis and stimulation of glucose uptake through translocation of glucose transporter type 4 (GLUT4) to the cell membrane, which is responsible for glucose uptake in muscle and fat (21).

Broadly speaking, endogenous insulin mediates metabolic activities via the AKT/protein kinase B (PKB) metabolic pathway (23). Additionally, sustained IR stimulation can induce a mitogenic response via the mitogen-activated protein kinase (MAPK) pathway (23). This pathway plays a minor role, if any, with endogenously secreted insulin since serum concentrations are generally low and highly regulated; however, therapeutic insulin, specifically insulin analogues, require greater consideration of the MAPK pathway and the related insulin-like growth factor-1 (IGF-1) receptor signaling pathway. Thus, it is important that any new insulin be characterized to ensure the metabolic and mitogenic signaling properties are appropriate, relative to native insulin (24).

**Figure 3.**
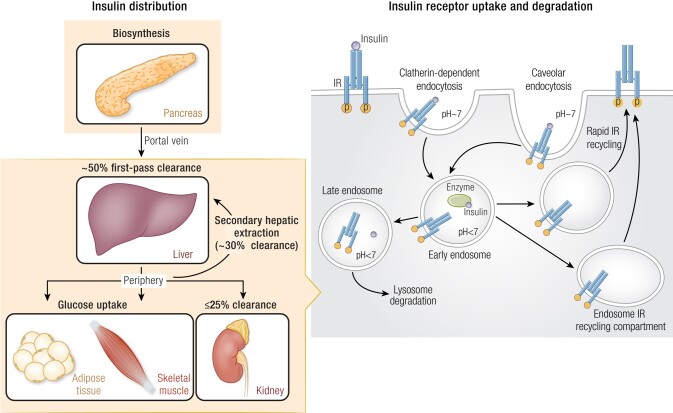
Metabolic pathway for endogenous insulin. Left, The distribution of endogenous insulin through the body. Endogenous insulin is produced in the pancreas. It is then transported to the liver through portal circulation. The majority of insulin (40%-80%) is cleared by the liver by hepatocytes with approximately 50% cleared through first-pass extraction from the portal vein. The insulin exiting the liver is distributed to the adipose tissue muscle and kidney, where it controls the utilization of glucose and free fatty acids for energy. Any insulin that is not distributed to the parenchyma is either filtered by the kidney (∼25%) or recycled back to the liver by the arterial blood flow, where an additional approximately 30% is cleared. Right, Generalized mechanism for insulin intracellular degradation via IR-mediated endocytosis in the liver. Insulin binds to the IR and forms a complex inducing internalization into the cell via 2 routes, clatherin-dependent or caveolar endocytosis. Receptor-bound insulin is released in the acidic early endosome and is degraded by enzymes that include protein disulfide isomers, insulin-degrading enzyme, and cathepsin D. The IR is recycled back to the cell surface by the rapid IR recycling and endosome IR recycling compartment pathways. Any degraded IR and insulin fragments are routed to the lysosome for further degradation. IR, insulin receptor.

On binding to the receptor, phosphorylation of the β-subunit controls IR internalization and trafficking through receptor-mediated endocytosis (22, 23). In acidified intracellular endosomes, insulin is released from the IR, allowing various enzymes to degrade the hormone, most notably, insulin-degrading enzyme (IDE) (25), cathepsin D (26, 27), and protein disulfide isomerase (Fig. 3) (28). This postinternalization degradation in cells is the major pathway for insulin elimination. As discussed later, the reduced affinity of IR binding with once-weekly insulins and subsequent reduced postreceptor clearance is one mechanism by which the duration of action of these once-weekly insulins is prolonged.

#### Biology

The biology of insulin has been extensively reviewed (29). The energy demands of the human body (ie, adenosine triphosphate [ATP] production) throughout the day uses a variety of substrate sources (glucose, glycogen, fatty acids, ketones, and more rarely amino acids) depending on the presence of insulin and glucagon, hormones that facilitate energy-source storage and utilization (30). Insulin mediates numerous cellular effects on tissues including, but not limited to, muscle, adipose, liver, and kidney tissues. Notably, insulin controls the use and storage of (a) carbohydrates by increasing glucose uptake, enhancing glycolysis, driving glycogen synthesis, and attenuating glycogen breakdown; (b) lipids by attenuating lipolysis to regulate availability of free fatty acids, increasing triacylglycerol synthesis for triglyceride formation, increasing uptake of triglycerides from the blood, and attenuating fatty acid oxidation; and (c) proteins by enhancing uptake of some amino acids, accelerating protein synthesis in muscle, and downregulating protein degradation (31).

Consequently, in healthy individuals, insulin is continuously released from pancreatic β cells to maintain euglycemia by controlling both endogenous glucose production in the liver, and to a lesser extent from the kidneys, as well as exogenous glucose uptake from dietary sources during intermeal and mealtime periods (32). This insulin secretion is pulsatile with a frequency of these secretions occurring every 5 to 15 minutes (33-35). In the fasted state, insulin release is reduced and referred to as a basal profile. In the fed-state, insulin secretion is increased (bolus secretion) to attenuate hepatic glucose production (HGP) and increase glucose utilization. In healthy individuals, the pancreatic insulin demands to maintain euglycemia are parsed to approximately 50% for the basal periods and approximately 50% for postprandial periods (32). The pulsatile secretion of endogenous insulin is layered onto a circadian rhythm with the rate of insulin secretion rising during the morning hours, peaking in the afternoon, and then decreasing during the evening and when sleeping (34, 36). This circadian periodicity helps control endogenous insulin release to compensate for the effects of insulin counterregulatory hormone surges in the morning (eg, growth hormone and cortisol), while facilitating increased nocturnal HGP to compensate for reduced intermeal glucose levels.

#### Whole-Body distribution

In healthy individuals, 40% to 80% of the pancreatic-secreted insulin is used and cleared through the IR in hepatic tissues (see Fig. 3) (37-40). This level of insulin extraction by hepatic tissues is attributed to both first-pass extraction from the portal vein (∼50%) and secondary extraction from hepatic arterial blood supplies (∼30%) (41, 42). Specifically with first-pass extraction, the locally high insulin concentration, coupled with the high affinity of native insulin for the IR, ensures effective suppression of HGP by limiting glycogenolysis (43) and gluconeogenesis (44). The creation of this hepatic/peripheral insulin concentration gradient by hepatic uptake and clearance modulates insulin exposure to peripheral tissue relative to the liver (41, 42), thus controlling glucose uptake from the blood.

**Figure 4.**
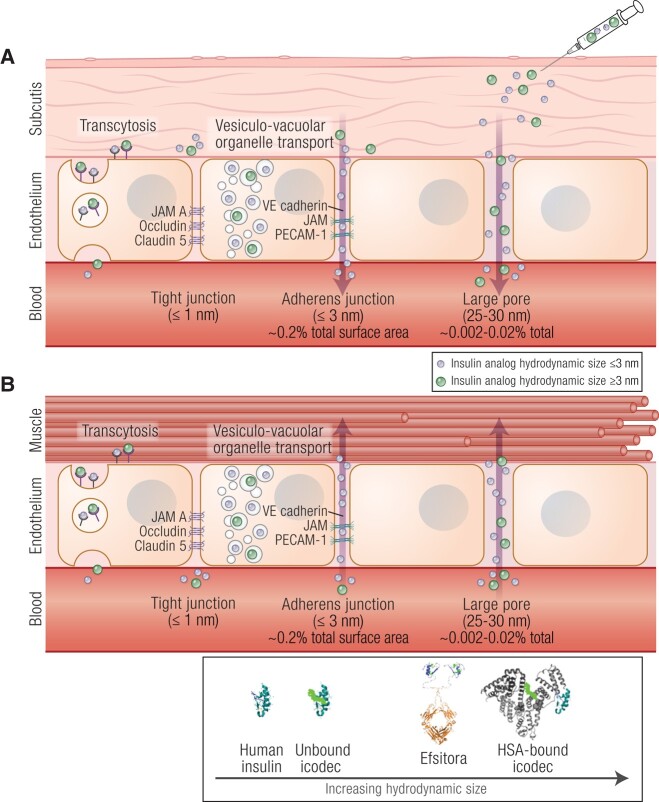
Pore theory of insulin transport pathways. Insulin molecules, based on their hydrodynamic size, can use multiple paths to reach circulation from A, the subcutaneous space and B, the parenchymal tissue from the circulation. Insulins may use vesicular-vacuolar organelle transport, or transcytosis through binding to insulin receptors for transcellular transport. Insulin molecules with a hydrodynamic size less than 3 nm such as human insulin and unbound icodec can also use adherens junctions for paracellular transport. Very large insulin molecules such as efsitora (molecular weight 64.1 kDa) and HSA-bound icodec (molecular weight ∼73 kDa) are thought to predominantly use the large pores (25-30 nm). Both are likely absorbed from the subcutaneous depot via the slow-flowing lymphatic system due to their large hydrodynamic size, which slows the release into the circulation and limits parenchymal exposure. HSA, human serum albumin.

Insulin exposure to parenchymal tissues (eg, adipose and muscle tissue) is controlled by paracellular junctions in the capillary endothelium (Fig. 4). The perfusion of these tissues is adequately described by “pore theory,” wherein the hydrodynamic size of insulin enables transport across the capillary endothelium. Transport across the capillary endothelium takes advantage of the high rate of filtration and reabsorption of fluid across adherens junctions, which are less than or equal to 3 nm in diameter and account for approximately 0.2% of the total surface area of the capillary endothelium and, to a lesser extent, large paracellular gaps, which are estimated to be 25 to 30 nm in diameter and account for 0.002% to 0.02% of the total surface area of the capillary endothelium (19, 29, 45). These latter large paracellular gaps should not be confused with the fenestrated sinusoidal endothelial of the liver and kidneys, which are gaps greater than 100 nm in diameter.

Lastly, insulin perfuses the kidney where approximately 25% of the extrahepatic insulin is cleared (46-48), making this organ the second most important organ for insulin clearance (Fig. 3). In the kidney, insulin filtration occurs in the proximal tubule by diffusion across the glomerular capillaries and is taken up by the IR in the peritubular capillaries. Insulin engagement of the IR in the kidney also produces a pleiotropic array of activities, including glucose uptake by podocytes, maintenance of barrier permeability, stimulation of glucose reabsorption, control of kidney gluconeogenesis, and insulin degradation (49). Notably, the clearance of insulin can be slowed in patients with diabetic nephropathy/chronic kidney disease (CKD), necessitating dose adjustment of some insulin therapies, such as human insulin and IGlar, if renal function deteriorates, as discussed later (50-52).

#### Clearance

Endogenous insulin has a biological half-life of 3 to 10 minutes (29, 53) or absolute clearance rate from the blood of 32 to 84 L/h (19), which is rapid and akin to glucagon-like peptide-1 (GLP-1), a small peptide with a clearance rate of 145 L/h (54). This rapid clearance and elimination of insulin from the circulation is directly linked to the distribution to tissues (liver, kidney, and parenchyma) where IR-induced endocytosis leads to rapid plasma clearance followed by intracellular enzymatic degradation (19, 25) (see Fig. 3).

### Exogenously Administered Once-Daily Basal Insulins

#### Structures and structural properties

While endogenous secretion and utilization of insulin is highly regulated in healthy individuals, people with T1D with marked insulin deficiency, and some people with T2D during later stages of the disease, are unable to meet all the insulin demands of the body, and specifically to this review, basal insulin demands. Consequently, this deficiency requires therapeutic insulin supplementation to maintain euglycemia. In this section, the characteristics, attributes, and limitations of currently available once-daily basal insulins are discussed.

The creation of a desirable therapeutic basal insulin needs to address, at least, 3 primary challenges: (a) duration of action, (b) day-to-day and/or within-day SC absorption variability, and (c) hypoglycemia risk, especially during the overnight hours.

**Figure 5.**
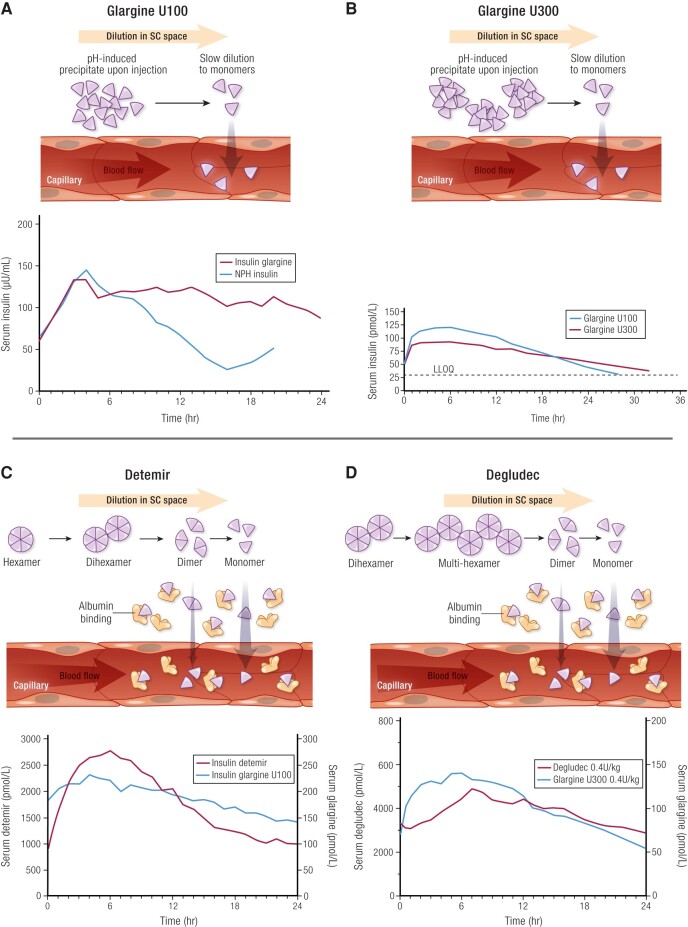
Daily basal insulin analogues. Adapted with permission from Hirsch et al (1). A, Left: Mechanism of protraction of IGlar U100 through pH-induced precipitation at the SC space. Right: PK profiles of IGlar U100 compared to NPH (each 0.3 U/kg) from a euglycemic clamp study in 20 individuals with T1D. Data from Lepore et al (55). B, Left: Mechanism of protraction of IGlar U300 through pH-induced precipitation at the SC space. Mechanism is the same as for IGlar U100 but with a more sustained release due to the more concentrated formulation resulting in slower release of insulin glargine from the precipitate. Right: PK profile of IGlar U300 compared to IGlar U100 (each 0.4 U/kg) from a euglycemic clamp study in 18 individuals with T1D. Data from Becker et al (13). C, Left: Mechanism of protraction of IDet through di-hexamer formation in the SC space and binding to albumin. Right: PK profile of IDet compared to IGlar U100 (both 0.35 U/kg) from a euglycemic clamp study in 12 patients with T1D. Data from Porcellati et al (58). D, Left: Mechanism of protraction of IDeg through sustained release from multihexamer formation and binding to albumin. Right: PK profile of IDeg compared to IGlar U100 (both 0.4 U/kg) in 22 patients with T1D. Data from Heise et al (11). NPH, neutral protamine Hagedorn; PK, pharmacokinetic; SC, subcutaneous; T1D, type 1 diabetes.

The first real breakthrough in addressing these challenges was the development of IGlar U100, an insulin analogue, which was approved in 2000 (1). This elegantly designed basal insulin used amino acid changes to shift the isoelectric point of insulin nearer to neutral pH. This shift allowed the preparation of IGlar in an acidic unbuffered solution that allowed for insulin precipitation at neutral pH in the SC depot (Fig. 5), slowing the release of insulin into the circulation for durations of time up to 18 to 24 hours in most patients and producing a therapeutic half-life of 12 to 15 hours (1, 19, 55). Being a solution, IGlar U100 did not require resuspension, unlike NPH and ultralente. This, together with its long half-life, provided extended glycemic control with less variability. In addition, the reduced P/T ratio of IGlar U100, when compared to NPH, lowered the risk of nocturnal hypoglycemia, providing a tangible benefit and clinical advance in basal insulin replacement (1).

A different strategy to extend the time-action profile and reduce variability of action was employed with IDet U100 approved in 2004 (Europe)/2005 (United States) and IDeg U100 and U200 approved in 2015 (1). Both products, which are also solution formulations, used a modified insulin (ie, des-B30), which was conjugated to an acyl chain at a lysine site (ie, Lys-B29) (56, 57). The acyl chains introduced 2 important time-action properties: specifically, higher-order hexamer association in the SC depot and reversible binding to human serum albumin (HSA) in the SC, serum, and interstitial fluids. IDet used a 14-carbon fatty acid chain while the second-generation IDeg incorporated a 16-carbon acyl diacid, which further extended the duration of action (56, 57). These modifications introduced higher level association in the SC depot, that is, dihexamerization of IDet hexamers (56) and multihexamerization of IDeg hexamers (57), which protracted release of monomeric insulin into the circulation (see Fig. 5). The reversible binding to HSA also creates a bound “reservoir” of nearly inactive insulin, which can reversibly dissociate from HSA to yield an acylated insulin derivative capable of engaging the IR.

The soluble nature of these acylated insulins, in both the formulation and in the SC depot, reduced variability relative to the first-generation basal insulins NPH and ultralente, which require resuspension prior to use. However, the short duration of action of IDet, ie, t_1/2_ = 5-7 hours, and clearance of 8.4 L/h, which is only approximately 10 times slower than endogenous insulin, necessitated twice-daily dosing in many patients to provide adequate daily basal insulin coverage, especially in those with T1D (1, 19, 58). The iteration to IDeg increased the duration of action beyond 1 day, that is, t_1/2_ = 25 hours and clearance of 2.1 L/h, which is up to 40 times slower than endogenous insulin and attributable, in part, to stronger binding to HSA (1, 19). Collectively, these attributes allowed IDeg the flexibility of injection any time in an 8- to 40-hour window at steady state without losing efficacy or accumulating insulin (59). In addition, IDeg showed lower glucose variability compared to IGlar U100 and consequently lowered the risk of hypoglycemia.

IGlar U300 was introduced in 2018, by creating a more concentrated formulation of insulin glargine, which altered precipitation properties in the SC depot and slowed insulin absorption thereby prolonging the half-life of IGlar U300 to 19 hours (see Fig. 5) (19). This advance was considered a clinically significant improvement over IGlar U100, again by contributing to lower glycemic variability and lower hypoglycemia risk compared to IGlar U100 (15, 16).

#### Signaling and biology

Exogenous basal insulin molecules, by design, were developed to mimic the insulin signaling and cellular biology observed with endogenous insulin, that is, to bind to the IR causing a series of phosphorylation events that mediate pleiotropic intracellular activities as discussed earlier. The first- and second-generation long-acting soluble basal insulins demonstrate binding affinities for the IR of approximately 50% of human insulin for IGlar (60) and approximately 15% of human insulin for IDet (61) and IDeg (62). Although weaker binding agonists, relative to human insulin, these basal insulins generate the same insulin signaling and cellular biology pathway activation observed with endogenous insulin (63).

The engineering/acylation of these exogenous insulins necessitate greater scrutiny of mitogenicity mediated by the MAPK pathway. Of particular importance is the mitogenic potential of nonnative basal insulin analogues, where the relative mitogenic-to-metabolic activity needed to be similar to native human insulin to prevent risk of cell proliferation (oncogenic risk) greater than native insulin (64). This was an unwarranted concern raised for IGlar U100 that was dissipated with the better understanding of the actions of IGlar metabolites M1 and M2 (65). The main consideration, however, remains, that the mitogenic potential of all insulin analogues is something that must be considered.

#### Whole-Body distribution

As with endogenous insulin, parenchymal tissues can be exposed to therapeutic basal insulins through paracellular junctions in the capillary endothelium as described by the pore theory outlined previously. Depending on their hydrodynamic size, some basal insulins (ie, human insulin, IGlar, unbound IDet, and unbound IDeg) can use adherens junctions less than or equal to 3 nm to cross the capillary endothelium, whereas larger molecules (ie, HSA-bound IDet, HSA-bound IDeg) are hypothetically limited to the less prevalent 25- to 30-nm large paracellular gaps (see Fig. 4) (19). Consequently, the basal insulin therapy employed dictates which paracellular junctions can be used and thus control peripheral exposure. However, it should be noted, that all of these insulin analogues can access tissues with fenestrated sinusoidal endothelia.

Administration of insulin by SC injection not only alters the plasma concentration and time-action, but also distribution of the hormone to hepatic and parenchymal tissues. Notably, most therapeutic insulins will distribute equally across hepatic and extrahepatic tissues; therefore, with therapeutic insulin, the periphery can experience relative overinsulinization and the liver underinsulinization (19). Consequently, patients can experience inadequate suppression of HGP with therapeutically administered insulins (66-71) This is in contrast to endogenous insulin in mammals, where insulin secretion is directly into the portal vein to initially perfuse the liver where approximately 50% is used to control HPG, thus minimizing systemic insulin concentrations through hepatic utilization coupled with intracellular degradation (41, 42). To achieve sufficient insulin activity at the liver without raising peripheral insulin levels excessively (with resulting risk of hypoglycemia), the next generation of once-weekly basal insulins need to control peripheral exposure to mimic the hepatic to peripheral gradient of endogenous insulin.

#### Clearance

As with endogenous insulin, the plasma concentration of exogenous basal insulins is highly linked to tissue distribution (liver, kidney, and parenchyma) and the associated IR-induced endocytosis and enzymatic degradation. However, in contrast to endogenous insulin, in which the majority of insulin clearance occurs in the liver (40), exogenous unmodified insulin and human NPH insulin are cleared primarily by the kidney (30%-80%) (72). With regard to acylated basal insulins, IDet and IDeg, renal clearance is minimized by reversible binding to HSA, which is not readily filtered by the kidney due to the size and negative charge; consequently, these acylated insulins increase their plasma concentration. However, as discussed earlier, acylated insulin distribution to the parenchyma is restricted to transport of the limited amount of unbound acylated insulin across the adherens junctions, or hypothetically, HSA-bound acylated insulin across the less prevalent large paracellular gaps. This begins to shift the hepatic/peripheral gradient back toward that observed with endogenous insulin (67-69, 73). When studied in patients with renal failure (including end-stage renal disease) no PK differences were seen for IDeg (74) or IDet (75). In contrast, the time-action and clearance of IGlar is completely dependent on controlling absorption from the SC and tissue distribution, which is similar to exogenous human insulin. Although the PK of IGlar has not been evaluated in renal failure, some studies with human insulin have shown increased plasma insulin levels in the setting of compromised renal function necessitating dose reduction, which may therefore also be necessary in patients on IGlar (76, 77).

### Limitations With Once-Daily Basal Insulins

#### Distribution challenges

The goal of basal insulin replacement therapy is to attempt to mimic endogenous basal insulin activity. As discussed, unlike endogenous human insulin, all currently available once-daily basal insulin analogues are administered in the SC space; consequently, the hepatic/peripheral concentration gradient generated with endogenous insulin secretion is lost (2, 41). This can lead to underinsulinization of the liver and challenges with effectively controlling HGP. Moreover, attempts to increase hepatic insulinization with human insulins and insulin analogues can be fraught with challenges of overinsulinization of the peripheral tissues, which can result in increased risk of hypoglycemia and weight gain (41). These limitations are particularly applicable to human insulin formulations (eg, NPH) and IGlar, whereas evidence suggests that acylated insulin molecules (eg, IDet and IDeg) may possess better hepatopreferential profiles (41, 67, 73, 78, 79).

Preclinical and clinical research over the past decade has highlighted the value of insulin analogues with enhanced hepatic insulinization (67, 69-71). Insulin analogues that exhibit a more hepatoselective profile with controlled peripheral exposure have the potential to mimic the hepatic/peripheral insulin concentration gradient seen with endogenous insulin (41). Insulin peglispro, a 25.8-kDa molecule consisting of a 20-kDa polyethylene glycol (PEG) chain covalently bound to lysine-B28 of insulin lispro, was developed to try to provide a more “physiological” insulin profile (80). The distribution properties of insulin peglispro mimic, to a degree, the hepatic/peripheral distribution gradient observed in normal physiology (80). The large hydrodynamic size of insulin peglispro slowed exposure to the parenchyma by limiting access to only large paracellular junctions (25-30 nm), while still allowing facile passage into the liver tissue via fenestrations (100-200 nm) in the hepatic sinusoidal endothelium. Insulin peglispro demonstrated a longer half-life (24-46 hours) and slower clearance (1.3 L/h), which was approximately 65 times slower than native insulin (19). Moreover, insulin peglispro demonstrated promising results by attenuating peripheral glucose uptake at comparable HGP suppression levels to IGlar U100 in healthy individuals (69), and patients with T1D (70, 80, 81). A pooled analysis of 5 clinical trials demonstrated reduced nocturnal hypoglycemia with insulin peglispro compared to IGlar (82). However, because of hepatic side effects, notably, increases in alanine transaminase (ALT), possibly related to hepatobiliary clearance of PEG, and an altered hepatic fat distribution profile relative to IGlar, the development of insulin peglispro was discontinued in 2015 (80).

#### Pharmacokinetic variability

The PK and PD variability of the once-daily basal insulins are, in part, affected by absorption differences from disparate SC depot sites, physical state of the insulin (ie, crystalline, amorphous precipitate, or solution), and frequency of dosing as a function of the half-life/clearance of the insulin. As described earlier, the current twice daily/once-daily basal insulin analogues have altered their rates of absorption either through precipitation and redissolution (IGlar) or via higher-order hexameric and HSA-association imparted by the addition of acyl chains (IDet and IDeg). Notably, IGlar U100 demonstrated more variability compared to IDet and IDeg (83, 84), due, in part, to redissolution of the precipitated/insoluble state of insulin in the SC. Furthermore, as the half-life of the basal insulin is prolonged, the P/T ratio can be reduced by enabling therapeutic accumulation (9, 85). These longer-half lives and reduced P/T ratios can lessen the effect of missed dosing and double dosing on PK profiles as described in Fig. 2B. With once-daily basal therapies with exposure profile of less than 1 day, each injection presents the patient with variation that is independent from previous injections; however, therapies that have longer half-lives than dosing frequency, such as IDeg, can average variability from prior injections allowing the patient to buffer the stochastic nature of an individual absorption process (Fig. 5D).

### Attributes of an Ideal Basal Insulin

Theoretically, an ideal basal insulin therapy may (a) possess a PK profile that continuously controls basal glucose production (more physiological); (b) possess a PK and PD profile that minimizes day-to-day variability (more predictable); (c) mimic the hepatic/peripheral insulin gradient seen with endogenous insulin (more physiological), thus minimizing overinsulinization of the extrahepatic tissue and attenuating the risk of hypoglycemia (safer); (d) reduce the frequency of injections (greater acceptance, adherence, and persistence); (e) simplify dosing (greater adherence and persistence); and (f) be responsive to changes in glucose (more physiological). In the next section we will describe the technology used to develop once-weekly basal insulins to address some of the challenges observed with once-daily basal insulin analogues and assess which of the attributes of an idealized basal insulin these molecules can achieve.

## Development Principles for Once-Weekly Basal Insulin

The insights afforded to scientists from the development of chemically modified insulins, that is, IGlar, IDet, IDeg, insulin-327 and insulin-406 (Novo Nordisk), and insulin peglispro, have guided engineering strategies that have produced 2 once-weekly insulin therapies in late-stage clinical development; insulin icodec (icodec or IDec; Novo Nordisk) and insulin efsitora alfa (efsitora or basal insulin Fc or BIF; Eli Lilly and Company) (86, 87). These molecules use similar strategies, but with some key differences, to extend basal activity. Most notably the attributes include (a) significantly attenuated IR binding affinity that appropriately modulates activation as a function of concentration and (b) secondary binding strategies to either HSA (icodec) or the neonatal Fc receptor (FcRn) (efsitora) to extend the time-action profile, slow clearance, and control tissue exposure. These characteristics appear to provide, ultra-long-acting basal insulins that could simplify patient usage and may contribute to improved adherence for patients.

Icodec recently completed an extensive phase 3 program (ONWARDS trials) (88) and has been submitted for regulatory review with first decisions anticipated in 2024 (89). Efsitora completed a phase 2 program and has commenced phase 3 trials (QWINT trials). Both insulins are designed for once-weekly administration and may use one-time loading-dose strategies; consequently, the molecules have the potential to introduce an advancement in basal insulin replacement, which was established over the past 20 years with the treat-to-target approach (4). The development principles underpinning these insulins are discussed next and then subsequently, emerging clinical data with once-weekly insulins are discussed.

### Prolonging Time-Action

Icodec and efsitora use multiple novel mechanisms to extend time-action.

#### Circulating “reservoir” of insulin for prolonging glucose-lowering activity

To date, a primary tool used for extending time-action is controlled SC release from the injection depot. As noted earlier with the once-daily acylated insulins, IDet and IDeg, hexameric and HSA association control the distribution of active monomeric insulin species that can cross the capillary endothelium to access peripheral tissues (90, 91). Moreover, binding to HSA minimizes both insulin activity and first-pass clearance by the kidneys.

**Figure 6.**
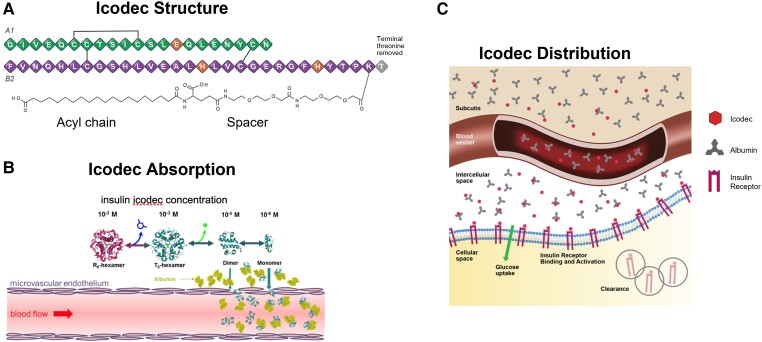
Insulin icodec. A, Icodec is an acylated insulin analogue with 3 amino acid changes (TyrA14Glu, TyrB16His, and PheB25His; orange) relative to human insulin to facilitate stability and reduce IR affinity. The reduced IR affinity tempers receptor-mediated clearance. A C20 icosane diacid is added with a spacer and enables strong and reversible HSA-binding to prolong plasma half-life. B, Delayed icodec absorption from the subcutaneous is achieved by diffusion controlled hexameric dissociation and binding of monomers to HSA. C, Icodec circulates primarily in an HSA-bound state with limited concentration of unbound icodec. The reduced insulin receptor affinity of icodec regulates binding to the IR by requiring higher local concentration for IR engagement; thus, providing more control of glucose uptake in the parenchyma. HSA, human serum albumin; IR, insulin receptor.

Interestingly, although once-weekly icodec is also an acylated insulin and forms hexamers, it deviates from its precursors (ie, IDet and IDeg) in that it does not form higher-order dihexamers or multihexamers (Fig. 6). Icodec protracts time-action through reversible, higher-affinity binding to HSA resulting in a large hydrodynamic size insulin/HSA complex that circulates systemically, creating a longer-lived reservoir in the blood for controlled active insulin generation for basal glucose control (see Fig. 6) (86, 92). The molecular weight of unbound (free) icodec is 6.4 kDa (93). The hydrodynamic size of free icodec is likely capable of using adherens junctions for absorption across the capillary endothelium from the SC space to the blood (Fig. 4A), and subsequent distribution to the parenchyma (Fig. 4B), whereas larger HSA-bound icodec (molecular weight ∼73 kDa) is unable to use these junctions. HSA-bound icodec, therefore, likely limits absorption and distribution through use of the less prevalent large paracellular junctions to cross the capillary endothelium, use of the lymphatic system (94), and/or by controlling the generation of unbound icodec.

**Figure 7.**
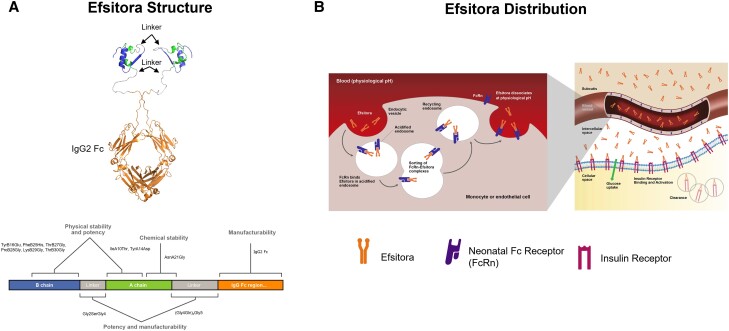
Insulin efsitora alfa. A, Efsitora is an insulin receptor agonist that is composed of a novel single-chain variant of insulin fused to a human IgG2 Fc domain. The insulin molecule has amino acid changes as shown in the figure to modulate IR affinity and reduce postreceptor clearance, as well as facilitate chemical stability and manufacturability. The reduced insulin IR affinity of efsitora regulates binding to the IR by requiring higher local concentration for IR engagement; thus, providing more control of glucose uptake in the parenchyma. B, Once injected, circulating efsitora binds to FcRn within the endothelial cells (insert). As seen in the insert, FcRn-bound efsitora is protected from degradation and is recycled back to the cell surface and into the blood. This creates a reservoir of insulin and prolongs circulating exposure. This protection/recycling system is controlled by pH switching where in the acidic endosome (∼pH 5.8) the Fc domain/FcRn binding is favored. However, at extracellular neutral pH environment such as in the blood (pH ∼7.2), efsitora release from the FcRn is favored. The reduced IR affinity of efsitora regulates binding to the IR by requiring higher local concentration for IR engagement; thus, providing more control of glucose uptake in the parenchyma. FcRn, Fc receptor; IR, insulin receptor.

The large hydrodynamic size of once-weekly insulin efsitora is achieved by fusion to an Fc domain (molecular weight of efsitora is 64.1 kDa) (Fig. 7) (87), which shifts absorption from the SC site to the slower flowing lymphatic system (95), and limits efsitora to the less prevalent large paracellular junctions to cross the capillary endothelium (see Fig. 4A and 4B). Additionally, efsitora makes use of the FcRn recycling system to prolong action. The Fc domain of efsitora binds to endogenous FcRn to extend exposure and protect the efsitora from elimination due to pinocytosis (96); which enables the creation of a systemic reservoir of available insulin for basal glucose control (see Fig. 7). Proteins in the blood, eg, immunoglobulin G (IgG), are susceptible to cellular uptake via pinocytosis, which is the process by which extracellular solutes are taken up into a cell via small vesicles. The FcRn system protects IgG, which contains an Fc domain, from degradation in the acidic vesicles created on pinocytosis and extends exposure by using pH-dependent recycling of the IgG back to the blood. This protection/recycling system is controlled by pH switching; that is, in the acidic vesicles (∼pH 5.8) the Fc domain/FcRn binding is favored and protection is afforded; however, dissociation is favored in the extracellular neutral pH environment (pH ∼7.2) allowing for recycling (see Fig. 7) (96). Fusion proteins, such as efsitora (87) and dulaglutide (97), incorporate this Fc domain to create a circulating reservoir of the therapeutic agent with long and continuous action by using the FcRn recycling system.

#### Clearance

As noted, therapeutic insulin is cleared from the blood by tissue distribution to the liver, kidney, and parenchyma, where IR-mediated endocytosis leads to insulin degradation. Consequently, slowing clearance from the body requires controlling tissue distribution, attenuating IR-mediated endocytosis, and limiting first-pass renal filtration.

#### Controlling tissue distribution

Although no formal insulin distribution studies have been reported to date for these 2 once-weekly insulins, much can be inferred from the literature.

Preclinical insights from insulin-327 and insulin-406, 2 acylated insulins with tight affinity for HSA that demonstrate hepatoselectivity in dogs (67, 98), coupled with clinical insights from IDet (73), suggest that the increased binding affinity of icodec to HSA could attenuate peripheral exposure and increase hepatic exposure.

Based on the evidence generated by research on antibodies (99-101) and antibody fragments of varying molecular weight (102), molecules akin to efsitora transit slowly across the vascular endothelium by convection through different sized pores in the vascular wall. Moreover, the concentration of hydrodynamically large and polar proteins in tissues is substantially reduced relative to plasma concentrations due to this slow convective uptake and rapid target-mediated elimination. Biodistribution studies with nonbinding IgG established the range of tissue-to-blood ratio at 0.004 to 0.68 (100, 101). In organs and tissues relevant to glucose control, antibody concentrations relative to plasma, were 14% and 12%, respectively in the kidney and liver, and 5% and 4%, respectively in adipose and muscle tissue (100, 101). In addition, studies with antibody fragments of varying molecular size show that molecules of approximately 60 kDa (eg, a single-chain biospecific antibody (scFv)_2_), akin to efsitora, have similar biodistribution to gluconeogenic organs, that is, the liver and kidney (102). Although no studies have yet definitely shown the tissue action profile for efsitora, the prospects of using a systemic depot system acting as a reservoir for efsitora, exploiting large paracellular junctions to regulate distribution to tissues, and attenuating IR engagement, may provide the desired control of peripheral insulinization to enable once-weekly administration.

#### Attenuating insulin receptor–mediated endocytosis

Weakening IR affinity, through appropriate protein engineering and acylation, can attenuate receptor-mediated clearance in insulin-sensitive tissue by increasing the local concentration requirement for IR engagement. This attenuated binding affinity, coupled with control of available active basal insulin distributed to extrahepatic tissue, governs IR activity and receptor-mediated endocytosis, and, by extension, insulin clearance and degradation.

**Figure 8.**
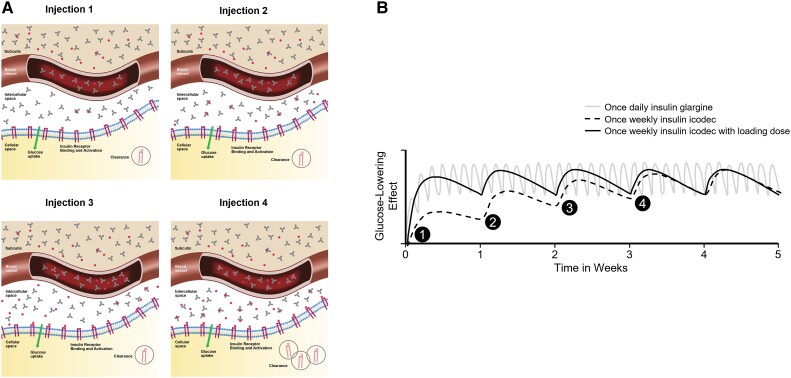
Icodec dosing and build-up to efficacious exposure. A, Schematic depiction of the distribution of insulin icodec (red hexagons) bound to albumin (gray) in the different biological compartments over time from initiation of once-weekly dosing (injection 1) through injection 5, showing the accumulation of insulin icodec in the intercellular space. B, Modeling of insulin icodec concentration when dosed without a loading dose (black dashed) and with a loading dose (black solid) compared to once-daily insulin glargine U100 (gray).

With icodec, reduced receptor-mediated clearance is achieved by using 3 amino acid substitutions (TyrA14Glu, TyrB16His, and PheB25His) to weaken IR affinity as well as improve stability (see Fig. 6). Affinity modulation, coupled with stronger albumin binding, using an icosane C20 fatty diacid, ensures the formation of a large reservoir of HSA-bound insulin in the blood and periphery that is available for the sustained release of active insulin, albeit with attenuated affinity (Fig. 8) (86, 92).

At the level of the receptor, icodec is more selective for the IR vs the IGF-1 receptor (IGF-1R) and once bound to the IR, icodec shows similar affinity for both IR isoforms A and B (86). The binding affinity of icodec for IR isoform A is 0.5% that of human insulin in the absence of serum albumin and 0.03% that of insulin when assessed in the presence of 1.5% HSA (86). In cell-based assays, icodec was a full agonist for the IR with a balanced mitogenic-to-metabolic potency ratio comparable to insulin as monitored by signaling (phosphorylation of IR, AKT/PKB, extracellular signal-regulated kinase), metabolic activity (lipogenesis and glycogen synthesis), and mitogenic activity (DNA synthesis). In addition to IGF-1R affinity, the IR (particularly IR isoform A) residence time has been implicated as a factor in the mitogenic potential of some insulin analogues (23). While the IR binding kinetics and residence time have not been reported for icodec, compared to human insulin, the in vitro mitogenic effects of icodec with respect to mitogenic activity in cells were categorized as low (86). Thus, icodec signaling properties are similar to native insulin; however, with reduced binding affinity. Despite reduced IR binding and weaker potency, icodec is a full agonist of the IR and elicits robust glucose-lowering capability (92).

Efsitora exists as a covalent homodimer with each monomer composed of a single-chain variant of insulin (SCI), wherein the B-chain is linked to the A-chain by a short linker and the SCI is linked to the Fc domain by an interdomain linker that connects the C-terminus of the SCI to the N-terminus of an IgG2 Fc domain (see Fig. 7) (87). Efsitora uses amino acid changes at TyrB16Glu, PheB25His, ThrB27Gly, ProB28Gly, LysB29Gly, ThrB30Gly, IleA10Thr, TyrA14Asp, and AsnA21Gly, coupled with the SCI format, to modulate IR affinity as well as contribute to manufacturability properties (eg, expression, chemical stability, and physical stability) (see Fig. 7) (87).

Preclinical data demonstrated that efsitora is a selective agonist for the IR vs IGF-1R (87). In IR binding assays, efsitora showed an approximately 100-fold reduced binding affinity compared to native insulin. In cell-based assays evaluating the functional activation of IR tyrosine autophosphorylation, efsitora had reduced potency for activation of IR, consistent with the binding data, and exhibits some degree of selectivity for activation of IR-B phosphorylation compared to IR-A, relative to native insulin. While the biological relevance of this is not clear, these data indicate that efsitora may have signaling selectivity for IR-B, the isoform associated with metabolic signaling, as opposed to IR-A, which is more associated with mitogenic signaling (87). Following activation of IR by efsitora, a more rapid dephosphorylation of the IR was observed compared to native insulin, suggesting that efsitora had a faster off rate from the IR and a favorable dephosphorylation profile relative to a mitogenic insulin analogue (AspB10) (87). In cell-based functional assays for metabolic (lipogenesis) and mitogenic potential, efsitora exhibits full IR agonism, however, with reduced potency compared to insulin, which is consistent with reduced IR binding affinity. Despite attenuated IR binding potency of efsitora, robust glucose-lowering efficacy with long duration of action is observed in vivo (87).

The attenuated IR binding and clearance in insulin-sensitive tissues allow accumulation to increase insulin concentrations of both icodec and efsitora explaining their robust glucose-lowering efficacy despite highly attenuated IR binding potency. However, since the tissue levels of both these insulins are not known, these assumptions remain speculative.

It is important to note that no mitogenicity concerns have been found in animal or in vitro preclinical studies for either icodec or efsitora, but, as for with any novel insulin, careful surveillance in real-world use will be required to develop full confidence in their safety.

#### Limiting first-pass renal filtration

First-pass renal clearance, via fenestrated endothelium, is a significant route of clearance for therapeutic-unmodified insulin, that is, human insulin or IGlar, which have molecular weights of ∼6 kDa. As noted earlier, the clearance of insulin can be adversely altered in patients with diabetic nephropathy/CKD. Thus, renal impairment necessitates dose adjustments with human insulin and IGlar as kidney function deteriorates (50-52). However, increasing the hydrodynamic size of the insulin, such as insulin peglispro (103), or binding acylated insulins to HSA, such as IDeg (104), can eliminate the need for insulin dose adjustments in diabetes patients with CKD. These findings are relevant to weekly basal insulins too and have been taken into consideration in their development (105). With icodec acylation, the linker (2xOEG-gGlu) and fatty acid moiety (C20 fatty diacid) were selected for stronger, yet reversible, HSA binding to attenuate the extent of renal clearance (92). With efsitora, the conjugation to an Fc domain creates a large molecule (64.1 kDa), akin to the size of HSA, that can also limit filtration through the renal glomeruli (87).

#### Effect of prolonging time-action

Collectively, the effect of controlling distribution, IR affinity, and renal clearance can prolong PK and glucose-lowering. With icodec, the time-action profile is extended in diabetic rats with a concomitant reduction in glycated hemoglobin A_1c_ (HbA_1c_) (92). Efsitora has also demonstrated an extended time-action and prolonged glucose-lowering profile in streptozotocin-treated diabetic rats (87). These long duration exposure profiles warranted study in humans with these molecules, which are discussed later.

### Minimizing Hypoglycemia Risk

While the extension of the time-action profile is necessary for a once-weekly basal insulin, the expectation of possible prolonged and/or recurrent hypoglycemia are concerns. Ideally, a glucose-sensing basal insulin, in which insulin activity is controlled by levels of circulating glucose, could alleviate or prevent hypoglycemia concerns. However, such insulins are not currently available. As such, any new insulin needs to be studied carefully to ascertain hypoglycemia risk.

**Figure 9.**
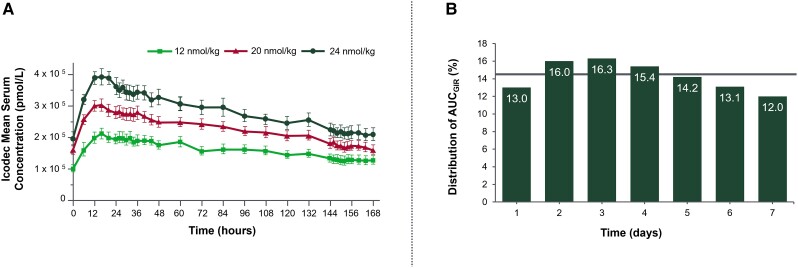
Pharmacokinetic and pharmacodynamic profiles of icodec in people with type 2 diabetes. A, Mean (SE) total serum icodec concentrations for 12, 20, 24 nmol/kg doses during week 5 of once-weekly dosing. PK results showed that icodec reached t_max_ at 16 hours after dosing, with a mean t_1/2_ of 196 hours. B: The PD effect of insulin icodec over a weekly dosing interval as derived from the observed data using a PK/PD model. The highest activity occurs at day 3 (∼16%), while on day 7 it is approximately 12%. An equal distribution across the 7 days of 14.3% per day is showed by the solid line. AUC_GIR_, area under curve for glucose infusion rate; HSA, human serum albumin; PD, pharmacodynamic; PK, pharmacokinetic; t_1/2_, half-life; t_max_, time to peak insulin concentration. Data from Nishimura et al (86).

Novo Nordisk has historically appeared to embrace a strategy designed to closely match insulin half-life to the desired dosing profile, for example, IDeg with a half-life of 25 hours, which is designed for once-daily dosing and icodec with a half-life of 196 hours (∼8 days) to support once-weekly therapy (19). This strategy enables faster attainment of steady state and faster reduction in plasma concentration post dosing, however, with an apparently slightly high P/T ratio. As illustrated in Fig. 8, steady-state concentrations can be achieved following 5 weekly doses of the same dose level. The time to steady state can be further accelerated by giving a one-time loading or starting dose (see Fig. 8). Although no P/T ratio has been reported for insulin icodec, based on the half-life, Heise (106) estimated the P/T ratio of icodec to be 1.81. PD modeling of icodec data shows that at steady state, over the course of 7 days, the highest activity occurs at day 3 (∼16%) while on day 7 it is approximately 12% (Fig. 9) (86). An estimate by Home (107) suggests the interday efficacy variability of icodec to be 1.36 on day 3 relative to day 7.

**Figure 10.**
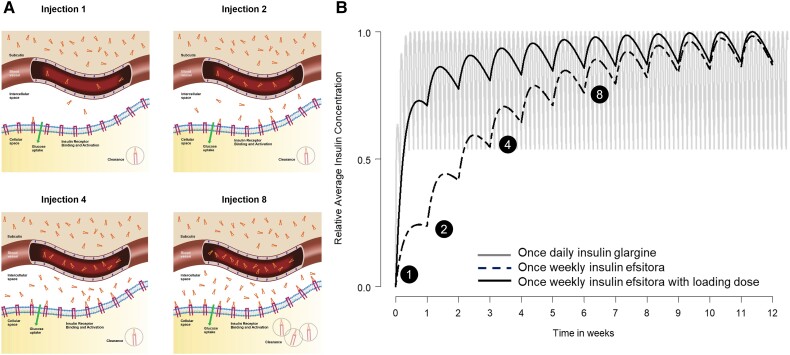
Efsitora dosing and build-up to efficacious exposure. A, Schematic depiction of the distribution of efsitora in the different biological compartments over time from initiation of once-weekly dosing (injection 1) through injection 8, showing the gradual movement of insulin efsitora from the subcutis through the blood to the intercellular space where build-up occurs. B, Model of insulin efsitora concentration when dosed without a loading dose (black dashed) and with a loading dose (black solid) compared to once-daily insulin glargine U100 (gray).

**Figure 11.**
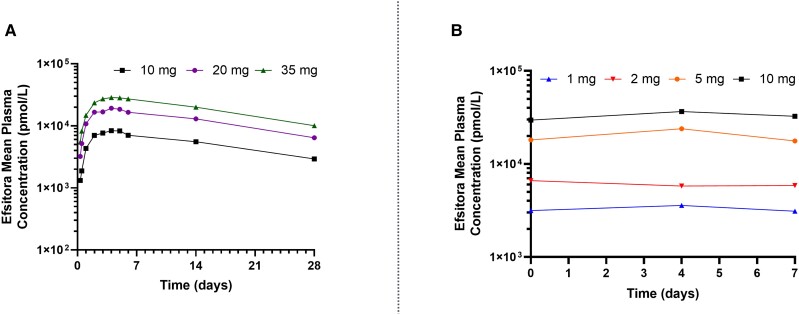
Pharmacokinetic properties of efsitora in people with type 2 diabetes. A, Mean plasma efsitora concentrations following a single subcutaneous dose (10, 20, and 35 mg doses) in people with T2D. PK results showed that efsitora reached t_max_ at 4 days after dosing, with a mean t_1/2_ of approximately 17 days. B, Mean plasma efsitora concentrations following dosing for 1, 2, 5, and 10 mg doses from a 6-week ascending dose study in people with T2D. The peak-to-trough ratio was determined to be 1.14. t_1/2_, half-life; t_max_, time to peak insulin concentration. Data from Heise et al (108).

The approach taken by Eli Lilly and Company with efsitora appears to embrace generation of the lowest P/T ratio. Efsitora has a relatively flat PK profile with an approximately 17 day half-life to support once-weekly dosing (Fig. 10). At steady state, efsitora has a P/T ratio of 1.14 (108) (Fig. 11). Time to steady state can be shortened by giving a one-time starting/loading dose (see Fig. 10). The long half-life of efsitora enables therapeutic accumulation and the generation of a low P/T ratio when administered weekly. Notably, the long half-life of efsitora could enable dosing intervals longer than 1 week; however, this would increase the required dose at each delivery, thus increasing the peak concentration and leading to a higher P/T ratio and may not necessarily simplify treatment as it is easier for patients to remember a weekly dose than a dose every other week.

Although ultimately hypoglycemia is caused by the mismatch between glucose levels and insulin availability, the PK data show that both icodec and efsitora have flatter insulin exposure profiles compared to once-daily basal insulins, which may translate to a day-to-day hypoglycemia risk that could be similar to or perhaps even potentially lower than once-daily basal insulins. Additionally, as discussed earlier, because of the large hydrodynamic size of HSA-bound icodec or efsitora, peripheral exposure and activity could be attenuated. It is important to note, however, that the effects of icodec or efsitora on hepatic glucose output relative to peripheral glucose uptake have not yet been studied and these theoretical attributes for hypoglycemia risk reduction with these molecules will need to be affirmed by robust clinical trial and real-world use data.

### Other Once-Weekly Basal Insulins in Development

Other than icodec and efsitora, there are several other molecules that have or are being studied as once-weekly basal insulins, all of which are either very early in development or have been discontinued. These have been described in a previous review (109).

## Emerging Clinical Data With Once-Weekly Insulins

### Insulin Icodec

#### Phase 1 studies

In a phase 1 clinical study in patients with T2D (n = 50), the median t_max_ of icodec was 16 hours and the mean half-life was 196 hours (∼8 days) (86). In this double-blind, double-dummy, randomized clinical trial, participants who were insulin-treated ± metformin received 5-week treatments of once-weekly icodec (12, 20, or 23 nmol/kg) plus once-daily placebo (n = 13, 13, 12) or once-daily IDeg (0.4 U/kg) plus once-weekly placebo (n = 12). At baseline, randomly assigned participants to receive icodec had a mean ± SD HbA_1c_ of 7.4 ± 0.6% and age 57.8 ± 4.3 years (86). On days 2 and 7 following the last insulin dose, PD properties at close to steady state were assessed in 24-hour glucose clamp procedures and the glucose-lowering effect over a once-weekly dosing interval was derived from the observed data using a PK/PD model. While the glucose-lowering effect (measured as a percentage of area under curve for glucose infusion rate [AUC GIR]) showed a close to even distribution over 7 days, there is a small increase from day 1 (13.0%) to day 3 (16.3%) and slight decrease on day 7 (12.0%) compared to day 3 (see Fig. 9) (86). The estimated difference suggests an equivalent of an approximately 36% higher effect seen on day 3 than day 7 as interpreted by Home (107). No serious or severe adverse events, severe hypoglycemic episodes, or injection site reactions were reported in this phase 1 study (86).

A second study investigated whether injection region affected exposure and glucose-lowering with icodec (110). Twenty-five participants with T2D received single SC icodec injections (5.6 U/kg) in the thigh, abdomen, or upper arm. Total icodec exposure, as measured by area under the curve from zero to infinity after a single dose, was similar between all 3 injection sites and the glucose-lowering effect coefficient of variation was also comparable at all injection sites (110).

#### Phase 2 studies

Dosing and titration strategies for icodec were tested to help inform phase 3 studies through a series of phase 2 studies, all in patients with T2D: 2 in insulin-naive patients (111, 112) and 1 in those already on once-daily basal insulin (113).

The first phase 2 icodec study was a 26-week study in 247 insulin-naive patients with T2D (111). Once-weekly icodec administered initially at 70 units (10 units × 7) was compared to once-daily IGlar U100 starting at 10 units. Both insulins were administered SC and titrated in a traditional treat-to-target approach to a fasting blood glucose target of 70 to 108 mg/dL. The primary end point was change in HbA_1c_ from baseline to week 26. Rosenstock et al (111) found that a baseline of HbA_1c_ of 8.1% and 8.0% with once-weekly icodec and IGlar U100 were reduced to 6.7% and 6.9% respectively, with an estimated between-group difference in HbA_1c_ change from baseline to week 26 of −0.18 percentage points favoring icodec (95% CI, −0.38 to 0.02; *P* = .080). There was a higher rate of level 1 (<70 mg/dL to ≥54 mg/dL) hypoglycemic events in the icodec group (5.09 events per patient-year of exposure [PYE]) compared with IGlar U100 (2.11 events per PYE; estimated rate ratio [ERR] 2.42; 95% CI, 1.50-3.88). However, the incidence of combined level 2 (<54 mg/dL) or severe (level 3) hypoglycemia was not statistically significantly different; 16.0% for icodec vs 9.8% for IGlar U100, with low rates of 0.53 and 0.46 events per PYE for icodec and IGlar U100, respectively (ERR 1.09; 95% CI, 0.45-2.65). There was only one participant that had an episode of severe hypoglycemia (defined as requiring assistance) in the icodec arm.

This study was followed by another, shorter study lasting 16 weeks, again in insulin-naive patients with T2D. In this study, icodec was dosed to 2 different fasting glucose targets: the American Diabetes Association (ADA)-recommended 80 to 130 mg/dL, and a more aggressive 70 to 108 mg/dL (112). Three titration algorithms with different once-weekly dosing were investigated. In the group with the ADA-recommended target (80-130 mg/dL), one protocol incorporated a weekly icodec increase or decrease (±) of 21 units (titration arm A) while the other used a ±28 unit change (titration arm B). The icodec arm with the more aggressive fasting glucose goal (70-108 mg/dL) was titrated once weekly with a ±28-unit change (titration arm C). The comparator was IGlar U100 titrated to a fasting glucose goal of 80 to 130 mg/dL with a weekly increase/decrease of 4 units. The investigators used percentage time in range (70-180 mg/dL) (TIR) during the last 2 weeks of treatment (weeks 15 and 16) as their primary outcome measure using a blinded Dexcom G6 real-time continuous glucose monitoring (rt-CGM). The results of the study showed that titration arm A (target 80-130 mg/dL and ±21-unit weekly icodec dose change) afforded the best balance between glycemic control while not increasing the risk of hypoglycemia compared to IGlar U100. Titration Arm B (target 80-130 mg/dL and ±28-unit weekly icodec dose change) showed a significantly greater TIR compared to IGlar U100 (estimated treatment difference [ETD] 7.08 percentage points; 95% CI, 2.12-12.04; *P* = .005) corresponding to an extra 102 minutes longer TIR. No severe hypoglycemic episodes occurred in any treatment group, and the rates of combined level 2 and level 3 hypoglycemia episodes were low for all insulin icodec titrations. Although overall hypoglycemia rates were low, rates of combined level 2 and level 3 hypoglycemia with icodec in titration arm B were higher compared to IGlar U100 (0.15 vs 0 events per PYE, respectively). Titration to attain a more stringent glucose target of 70 to 108 mg/dL (titration arm C) was also associated with a higher rate of hypoglycemia for icodec in comparison with IGlar U100, while TIR was not statistically significantly different. There was no clustering of level 1 hypoglycemic events in the days following the day of injection for icodec titrations A and B (glucose target 80-130 mg/dL), suggesting no noticeable “peak effect” with these approaches. The results of this study appear to support the titration algorithm used in the phase 3 trials where a fasting glucose target of 80 to 130 mg/dL was used with a ±20-unit weekly icodec titration (88).

As discussed earlier, a key difference between currently available once-daily basal insulins and once-weekly basal insulins is to determine if a one-time starting dose, or loading dose, is necessary to achieve an efficacious insulin steady-state level more quickly. This is particularly important for patients switching from once-daily basal insulins to once-weekly insulins to prevent transient hyperglycemia during the transition period. To test this hypothesis, a 16-week study investigated 2 approaches for switching to once-weekly icodec in 154 patients with T2D previously on basal insulin (113). Fifty-four patients were randomly assigned to a one-time starting dose of icodec calculated based on the previous basal insulin dose multiplied by 7 and then doubling this calculated dose as a one-time starting loading dose (total daily basal insulin dose × 7 × 2 [ie, 100% increase in the weekly insulin dose administered once at the beginning of the study]) followed by the participants going back 1 week later to their previously calculated total weekly dose (previous dose ×7) administered once a week as icodec. This loading-dose strategy was compared to 50 participants to whom no one-time loading dose was administered. The control group (also 50 individuals) received once-daily IGlar U100. All 3 groups were titrated to a fasting glucose target of 80 to 130 mg/dL with a ±28-unit weekly titration for icodec and ±4 units for IGlar U100. The primary outcome measure was percentage TIR (70-180 mg/dL) during the last 2 weeks of treatment (weeks 15 and 16) as measured by blinded CGM (Dexcom G6). The study showed a statistically significant difference in TIR favoring icodec when a loading dose was used (7.9 percentage points; 95% CI, 1.8-13.9) and no significant difference between icodec and IGlar U100 when there was no loading dose of icodec. Incidences and rates of level 1 hypoglycemic episodes were comparable between treatment arms and, while the rate and pattern of combined level 2 and level 3 hypoglycemic events appeared lower in the icodec treatment group with no loading dose than for the IGlar U100 group, these were similar between the icodec with loading dose and IGlar U100 groups. Time below range (TBR) (<70 mg/dL) was slightly higher for icodec with a loading dose (1.6%) compared to icodec with no loading dose (0.6%) or IGlar U100 (0.5%). Since the results of the study showed that the one-time starting-dose strategy was the most effective in increasing TIR and avoiding transient hyperglycemia, a loading-dose strategy, albeit with a lower loading dose of an additional 50% instead of 100%, was employed in the phase 3 icodec program for patients switching from a once-daily to a once-weekly basal insulin (88).

Overall, across the phase 2 studies, icodec achieved similar glycemic control to IGlarU100 (111-113). The rates of combined level 2 and level 3 hypoglycemic episodes were low for all treatment groups. In addition, a post hoc analysis of data from 2 of the phase 2 studies also found that hypoglycemia duration was similar with icodec compared to IGlar U100 in insulin-naive and insulin-treated patients with T2D, regardless of titration algorithm or use of a loading dose (114).

#### Phase 3 studies

Icodec's phase 3 program, entitled ONWARDS, consisted of 6 clinical trials. Key design features of the ONWARDS trials are outlined in Table 1 and described in detail by Philis-Tsimikas et al (88). ONWARDS 1 to 5 were treat-to-target studies in people with T2D, which assessed efficacy and safety of icodec compared to a once-daily comparator (IGlar U100 or IDeg) and/or placebo in combination with noninsulin glucose-lowering medications. ONWARDS 1, 3, and 5 were in insulin-naive patients. ONWARDS 2 and 4 were in insulin-treated populations, the former in patients on basal insulin and the latter in the setting of basal-bolus therapy. ONWARDS 6 was a treat-to-target study conducted in people with T1D in which the comparator insulin was IDeg.

**Table 1.** Icodec phase 3 trial design (ONWARDS 1-6)

	ONWARDS 1	ONWARDS 3	ONWARDS 5	ONWARDS 2	ONWARDS 4	ONWARDS 6
Clinicaltrials.gov No.	NCT04460885	NCT04795531	NCT04760626	NCT04770532	NCT04880850	NCT04848480
Population	T2D, insulin naive			T2D, previously insulin-treated		T1D
Study status	Complete					
Key trial details						
**Primary objective**	**Noninferiority in HbA_1c_ change from baseline compared to once-daily comparator**					
Key secondary assessments	Superiority in TIR (70-180 mg/dL); superiority in HbA_1c_ change; rate and incidence of level 2 and 3 hypoglycemia events	Superiority in HbA_1c_ change; rate and incidence of level 2 and 3 hypoglycemia events	PRO measures; rate and incidence of level 2 and 3 hypoglycemia events	Superiority in HbA_1c_ change; TIR (70-180 mg/dL); PRO measures; rate and incidence of level 2 and 3 hypoglycemia events	Superiority in HbA_1c_ change; TIR (70-180 mg/dL); rate and incidence of level 2 and 3 hypoglycemia events	TIR (70-180 mg/dL); rate and incidence of level 2 and 3 hypoglycemia events
Randomized trial design	Open-label	Double-blind	Open-label with real-world elements	Open-label	Open-label	Open-label
No.	984	588	1085	526	582	583
Trial duration, wk	78	26	52	26	26	52
Main phase	52	26	52	26	26	26
Extension phase	26	—	—	—	—	26
Once-daily comparator	Glargine U100	Degludec	Degludec, Glargine U100, or Glargine U300	Degludec	Glargine U100	Degludec
Bolus insulin during study	—	—	—	—	Aspart 2-4× daily	Aspart ≥2× daily
Background medications	Noninsulin glucose-lowering agents			± noninsulin glucose-lowering agents		−
Technology employed in study	CGM	—	Digital dose titration app	CGM	CGM	CGM
Key inclusion criteria						
Demographics	Adults aged ≥ 18 y					
HbA_1c_ at screening, % (mmol)	7.0-11.0 (53.0-96.7)	7.0-11.0 (53.0-96.7)	>7.0 (>53.0)	7.0-10.0 (53.0-85.8)	7.0-10.0 (53.08-85.8)	<10.0 (<85.8)
BMI	≤40.0	≤40.0	—	≤40.0	≤40.0	—

**Abbreviations:** aspart, insulin aspart; BMI, body mass index; MDI, multiple daily injections; PRO, patient-reported outcome; T1D, type 1 diabetes; T2D, type 2 diabetes; TIR, time in range.

In ONWARDS 1 to 4, and 6, insulin doses were titrated to a prebreakfast glucose target of 80 to 130 mg/dL with a weekly adjustment of ±20 U for icodec and ±3 U for the once-daily comparator (88). In ONWARDS 5, titration of icodec was guided by a digital app based on the titration algorithms used in the other ONWARDS studies while the once-daily basal insulin comparator (IDeg, IGlar U100, or IGlar U300) was chosen and titrated to standard of care at the discretion of the investigator (88, 115).

In the insulin-naive studies (ONWARDS 1, 3, 5), the starting insulin dose was 70 U per week for icodec (115-117). In ONWARDS 1 and 3, the starting dose for the once-daily comparator was 10 U per day and in ONWARDS 5, the comparator was initiated in accordance with local product labels. In the basal switch studies (ONWARDS 2 and 4), icodec starting doses were calculated as the pretrial total daily insulin dose multiplied by 7 (118, 119). For the first injection only, an additional 50% of this calculated once-weekly dose was given as a one-time loading dose before reverting to the standard weekly dose on week 2 with titration beginning the subsequent week (week 3). In the T1D study (ONWARDS 6), the weekly dose was calculated in the same way (daily basal dose times 7) and a one-time loading dose was given (88). This loading dose was either an additional 50% or 100% of the calculated starting dose depending on screening HbA_1c_ level (< 8.0% or ≥ 8.0%, respectively) or prestudy insulin treatment (ie, 50% one-time additional dose for participants previously receiving twice-daily basal insulin or IGlar U300, regardless of screening A_1c_). CGM data were collected using the Dexcom G6 system worn intermittently in blinded mode for ONWARDS 1, 2, 4 and throughout the study unblinded for ONWARDS 6 (88).

All studies achieved their primary end points of noninferiority to the once-daily comparator for HbA_1c_ change from baseline (noninferiority margin: 0.3%) (115-120). ONWARDS 1, 2, 3, 5 also achieved statistically significant superiority in HbA_1c_ reduction (115-118).

#### Studies in insulin-naive patients with type 2 diabetes

All 3 studies in insulin-naive patients (ONWARDS 1, 3, and 5) demonstrated statistical superiority of once-weekly icodec vs once-daily basal insulin comparators in HbA_1c_ reduction (115-117). In ONWARDS 1, mean HbA_1c_ was reduced from 8.5% or 8.4% at baseline to 6.9% and 7.1% at week 52 for icodec and IGlar U100, respectively, with an ETD for HbA_1c_ change of −0.19 percentage points (95% CI, −0.36 to −0.03), which confirmed noninferiority and superiority of icodec to IGlar U100 (116). TIR (70-180 mg/dL) at weeks 48 to 52 was significantly higher with icodec (71.9%) compared to IGlar U100 (66.9%; ETD 4.27 percentage points; 95% CI, 1.92-6.62). These statistically significant TIR differences were maintained through the extension phase (weeks 74-78) of the trial (116). Time above range (TAR; > 180 mg/dL) was statistically significantly lower with icodec (27%) compared with IGlar U100 (32%; ETD −4.58 percentage points; 95% CI, −6.99 to −2.17) at weeks 48 to 52. Notably, the superior HbA_1c_ and TIRs with icodec compared with IGlar U100 were demonstrated despite similar fasting plasma glucose (FPG) values in the 2 groups in this treat-to-target trial. These results raise the possibility that although FPG may still be appropriate to use for titrating weekly insulins, CGM metrics might be more informative in monitoring response to therapy with weekly insulins, a concept discussed further in the Clinical Implications Section. Similar findings were observed in the double-blind ONWARDS 3 study, which compared icodec to IDeg over 26 weeks. In ONWARDS 3, icodec demonstrated a statistically superior HbA_1c_ change from baseline to week 26 (ETD −0.2 percentage points; 95% CI, −0.3 to −0.1) compared to IDeg, again with similar FPG changes from baseline in the 2 treatment groups (ETD 0; 95% CI, −6 to 5 mg/dL) (117). ONWARDS 5 compared icodec titrated with a cloud-based dosing app with investigator-chosen daily basal insulin analogues (IDeg, IGlar U100 or IGlar U300) titrated at the investigator's discretion according to standard practice. In this study with some real-world elements, mean HbA_1c_ was reduced from 9.0% or 8.9% at baseline to 7.2% and 7.6% at week 52 for icodec with the app and once-daily analogues, respectively. An ETD for HbA_1c_ change of −0.38 percentage points (95% CI, −0.66 to −0.09) confirmed noninferiority (*P* < .001) and superiority of icodec with the app (*P* = .009) (115).

Overall level 2 (<54 mg/dL) hypoglycemia rates were low (<1 event per PYE) in all of these studies in insulin-naive patients. No episodes of severe hypoglycemia were reported for icodec in ONWARDS 3 and 5, and 1 episode was reported in ONWARDS 1 vs 7 with IGlar U100 (115-117).

In ONWARDS 1, at week 52 rates of combined clinically significant or severe hypoglycemia with icodec were 0.30 events per PYE compared with IGlar U100 at 0.16 events per PYE (ERR 1.64; 95% CI, 0.98-2.75) (116). When the 26-week extension phase and 5-week follow-up period were included, that is, at week 83, combined clinically significant or severe hypoglycemia rates were significantly higher with icodec (0.30 events per PYE) compared with IGlar U100 (0.16 events per PYE; ERR 1.63; 95% CI, 1.02-2.61) but still less than 1 event per PYE. The increased frequency of combined level 2 and level 3 hypoglycemia translated to 1 extra hypoglycemic event every 3 years. There was no significant difference in TBR (<54 mg/dL) at weeks 48 to 52 with icodec, (0.3%) compared with IGlar U100 (0.2%; estimated treatment ratio [ETR] 1.27; 95% CI, 0.94-1.71); both groups were below the guideline-recommended threshold of less than 1%. Significantly more icodec-treated individuals were able to achieve guideline target HbA_1c_ of less than 7% without level 2 or 3 hypoglycemia compared to IGlar (53% vs 43% at week 52; odds ratio [OR] 1.49; 95% CI, 1.15-1.94).

In ONWARDS 3, while a greater proportion of participants on icodec achieved a guideline HbA_1c_ target of less than 7% without level 2 or 3 hypoglycemia compared to IDeg (52% vs 40%), in contrast to ONWARDS 1 where the comparator was IGlar U100, there were almost 3 times more events of combined level 2 or 3 hypoglycemia with icodec compared to IDeg (50 events vs 17 events, respectively). Combined level 2 or 3 hypoglycemic rates were also statistically significantly higher from week 0 to week 26 in the icodec group (0.35 vs 0.12 events per PYE; 95% CI, 1.30-7.51; *P* = .01), all events being driven by level 2 hypoglycemia with no episode of severe hypoglycemia reported (117). These differences might be related to the considerably lower rate of hypoglycemia with IDeg in this study. As discussed earlier, IDeg has shown lower hypoglycemia risk compared to IGlar U100 (10).

In ONWARDS 5, in the setting of a significant HbA_1c_ difference in favor of icodec with the app, rates of combined level 2 or 3 hypoglycemia were not statistically significantly different (0.19 vs 0.14 events per PYE; ERR 1.17; 95% CI, 0.73 to 1.86) but numerically slightly higher with icodec with the app compared with once-daily analogues (115). A greater proportion of individuals on icodec with the app in ONWARDS 5 achieved a guideline HbA_1c_ target of less than 7% without level 2 or 3 hypoglycemia compared to once-daily insulins (41% vs 32%). It is noteworthy that in this study, patients were on significantly higher doses for icodec with the app vs once-daily analogues (227 vs 185 U/week; ETR 1.22; 95% CI, 1.12-1.33). The authors also note that no plateau was observed in icodec dose over the 52 week study period, whereas when dose adjustments were made by investigators according to standards of care in the once-daily analogues group, a plateau in insulin dose was observed around week 22. These results indicate that a supporting titration app could address the lack of titration often seen in clinical practice.

Although these hypoglycemia data in insulin-naive people with T2D are reassuring with a low frequency of events, particularly given that all 3 studies showed statistical superiority with regard to HbA_1c_ reduction, icodec-treated patients generally had higher event rates of hypoglycemia, especially when compared to IDeg, and caution would be appropriate in the less meticulously monitored real-world use until health care providers and patients accrue more experience with this therapy.

Another concern with the initiation of insulin therapy is weight gain. In ONWARDS 1, 3, and 5, modest increases in body weight were observed with icodec (2.2-2.8 kg); however, there were no significant differences between icodec and the once-daily insulin comparators (115-117). The weight gain in these studies was similar to that observed in the earliest studies with IGlar (4) as well as in a large observational study (121), and occurred in the setting of superior HbA_1c_ reduction and with similar, or in ONWARDS 5, higher, total insulin dose of icodec vs once-daily insulin comparators.

Since weekly insulins may help improve adherence to treatment, it is important to gauge patient preference. In ONWARDS 5, patient-related outcome measures were studied (115). The change from baseline to week 52 in the Diabetes Treatment Satisfaction Questionnaire (DTSQ) total treatment satisfaction score (ETD 0.78; 95% CI, 0.10-1.47) and the Treatment Related Impact Measure for Diabetes (TRIM-D) compliance domain score at week 52 (ETD 3.04; 95% CI, 1.28-4.81) statistically significantly favored icodec with the app compared with once-daily analogues. These findings could indicate greater patient acceptance of icodec with the app compared with once-daily basal insulins and its potential to address the challenges of inadequate titration and poor treatment adherence.

#### Basal insulin-switch studies in patients with type 2 diabetes

ONWARDS 2 was a 26-week study that investigated icodec compared with once-daily IDeg in patients with T2D, inadequately controlled on once-daily or twice-daily basal insulin (118). Mean HbA_1c_ was reduced from 8.2% or 8.1% at baseline to 7.2% and 7.4% at week 26 with icodec and IDeg, respectively, with an ETD of −0.22 percentage points (95% CI, −0.37 to −0.08), confirming noninferiority (*P* < .0001) and superiority (*P* = .0028) of icodec to IDeg. There were no statistically significant differences in TIR (70-180 mg/dL) or TAR (>180 mg/dL) for icodec vs IDeg assessed from week 22 to 26, with neither group achieving guideline recommended targets of more than 70% TIR (70-180 mg/dL) or less than 25% TAR (>180 mg/dL). The superior HbA_1c_ results for icodec were shown despite both TIR (70-180 mg/dL) (ETD 2.41 percentage points; 95% CI, −0.84 to 5.65; *P* = .15) and FPG change (ETD 0.71 mg/dL; 95% CI, −5.12 to 6.54; *P* = .81) being similar at study end for both treatment arms.

At week 26 the rates of combined level 2 and level 3 hypoglycemia were 0.73 events per PYE for icodec and 0.27 for IDeg (118). Overall rates of level 2 or level 3 hypoglycemia were numerically but not statistically significantly higher with icodec vs IDeg (ERR 1.93 events per PYE; 95% CI, 0.93-4.02) and more patients on icodec achieved a guideline-recommended HbA_1c_ target of less than 7% without experiencing level 2 or level 3 hypoglycemia (37% vs 27%). When assessed by CGM metrics at week 22 to 26, TBR (<54 mg/dL) was similar for both treatment arms and within guideline recommendation of less than 1%: 0.3% for icodec and 0.2% for IDeg (ETR 1.37; 95% CI, 0.92-2.04; *P* = .12).

There was a modest increase in body weight from baseline to week 26 associated with icodec, with an estimated mean change of +1.4 kg for icodec and −0.3 kg for IDeg (ETD 1.70; 95% CI, 0.76-2.63) in the setting of a higher total dose of icodec compared to IDeg at study end (268 vs 244 U/wk) (118).

Similar to that observed in the insulin-naive ONWARDS 5 study, the patients in ONWARDS 2, who were already on basal insulin at study entry, also appeared to show a preference for icodec vs IDeg based on significantly higher DTSQ total treatment satisfaction scores (ETD 1.25; 95% CI, 0.41-2.10; *P* = .0035) (118). These results could again suggest the potential for greater patient acceptance of weekly insulin therapy. However, these DTSQ data are from open-label studies and there may be a bias given the unblinded treatments. Additionally, the clinical trial setting may not fully reflect the preferences of individuals in the real world.

ONWARDS 4 was a 26-week study that investigated icodec compared with once-daily IGlar U100 in participants with T2D inadequately controlled on a basal-bolus insulin regimen (119). Mean HbA_1c_ was reduced from 8.3% at baseline to 7.1% at week 26 in both insulin arms. There was an ETD for HbA_1c_ change of 0.02 percentage points (95% CI, −0.11 to 0.15; *P* < .0001), which demonstrated noninferiority of icodec to IGlar U100. For weeks 22 to 26, TIR (70-180 mg/dL) was similar between icodec and IGlar U100 (67% vs 66%) and the TAR (>180 mg/dL) was also similar (30.5% vs 31.3%). These metrics did not reach guideline recommendations of more than 70% TIR (70-180 mg/dL) and less than 25% TAR (>180 mg/dL). FPG change from baseline to week 26 was similar between the 2 treatments (ETD −2.48 mg/dL; 95% CI, −10.59 to 5.63; *P* = .55).

There were significantly higher rates of level 1 hypoglycemia with icodec compared with IGlar U100 (31.5 vs 24.9 events per PYE; ERR 1.25; 95% CI, 1.03-1.52; *P* = .025) (119). Rates of combined level 2 or 3 hypoglycemia, however, were similar between icodec and IGlar U100 (5.6 vs 5.6 events per PYE; ERR 0.99; 95% CI, 0.73-1.33; *P* = .93). Across the trial period, there was no apparent clustering for combined level 2 or level 3 hypoglycemic events at any time point in the icodec or IGlar U100 groups nor was there any difference in nocturnal hypoglycemic events. There were 7 severe hypoglycemic events with icodec compared to 3 in the IGlar U100 arm. There were no significant differences between icodec and IGlar U100 for TBR (<54 mg/dL), which were within guideline recommended targets of less than 1%.

The authors found that although total dose increased for both groups, as would be expected in a treat-to-target trial, the total dose for the icodec group was significantly lower compared to the IGlar U100 group from week 24 to 26 (514 vs 559 U/week [∼73 vs ∼80 U/day]; ETR 0.92; 95% CI, 0.85-0.99; *P* = .034) (119). The authors further determined that this lower total dose was driven by a lower mealtime dose of aspart (not by the frequency of mealtime injection) and, interestingly, with the basal icodec dose being higher compared to IGlar U100 (305 vs 279 U/wk [∼44 vs ∼40 U/day]; ETR 1.09; 95% CI, 1.01-1.18; *P* = .029). Despite the differences in total dose of insulin between the groups, mean increases in body weight change from baseline were similar between icodec (2.7 kg) and IGlar U100 (2.2 kg; ETD 0.57 kg; 95% CI, −0.39 to 1.54; *P* = .34).

A post hoc analysis of TIR metrics from ONWARDS 2 and 4 (both studies conducted in patients previously on insulin) compared TIR metrics at the time of the switch to icodec (0-4 weeks) and again at steady state (22-26 weeks) (122). There was no difference in TIR metrics between the groups at the time of the switch. At steady state, again, both the icodec and comparator insulin groups showed similar improvement in TIR and TAR with no statistically significant differences between treatment arms. The TBR results were also similar except for ONWARDS 2 where there was a statistically higher TBR (<70 mg/dL) for icodec compared to IDeg (ERR 1.59; 95% CI, 1.21-2.08; *P* = .001) but not for TBR (<54 mg/dL) (122). Further analysis of these data found that duration of hypoglycemic episodes of less than 70 mg/dL were also similar with icodec vs IDeg or IGlar U100 during switch and at steady state (123). When these findings are viewed in conjunction with other published data from these studies that showed no clustering of hypoglycemic events at any time during the duration of the trials, the data are reassuring since they do not indicate that hypoglycemia risk is increased when a loading dose is administered.

#### Study in patients with type 1 diabetes

ONWARDS 6 was a 52-week study comparing icodec and IDeg in participants with T1D (main phase 26 weeks) (88, 120). In ONWARDS 6, mean HbA_1c_ was reduced from 7.59% at baseline to 7.15% at week 26 with icodec and from 7.63% to 7.10% with IDeg (120). The ETD for HbA_1c_ change was 0.05 percentage points with 95% CI, −0.13 to 0.23, which demonstrated noninferiority of icodec to IDeg (*P* = .0065). The change in mean HbA_1c_ from baseline to week 52 was statistically significantly lower with icodec than IDeg (−0.37 vs −0.54 percentage points; ETD 0.17 percentage points; 95% CI, 0.02-0.31; *P* = .021). For weeks 22 to 26, TIR (70-180 mg/dL) and TAR (>180 mg/dL) were similar between treatment groups with neither group achieving guideline-recommended targets of greater than 70% TIR (70-180 mg/dL) or less than 25% TAR (>180 mg/dL). Mean change in FPG from baseline to week 26 was lower with icodec (−15.1 mg/dL) vs IDeg (−33.7 mg/dL; ETD 18.6 mg/dL; 95% CI, 8.6-28.6], *P* = .0003).

The overall rates of combined level 2 or 3 hypoglycemia from baseline to week 26 were statistically significantly higher with icodec vs IDeg (19.93 vs 10.37 events per PYE; ERR 1.89; 95% CI, 1.54-2.33; *P* < .0001) (120). This significantly higher rate of combined level 2 or 3 hypoglycemia with icodec was maintained when the 26-week extension phase and 5-week follow-up period were included (ie, evaluation over 57 weeks). Rates of nocturnal combined clinically significant or severe hypoglycemia were also statistically significantly higher with icodec vs IDeg. For weeks 22 to 26, TBR (<54 mg/dL) was statistically significantly higher with icodec vs IDeg (1.0% vs 0.7%; *P* = .0014).

The mean weekly total insulin dose, adjusted for screening dose, was not statistically significantly different between icodec and IDeg from week 24 to 26 (311 U/wk [∼44 U/d] vs 323 U/wk [∼46 U/d]; ETR: 0.96; 95% CI, 0.90-1.03; *P* = .27) (120). The mean basal insulin dose was statistically significantly higher for the icodec group compared to the IDeg group from week 24 to 26 (170 U/week [∼24 U/d] vs 151 U/wk [∼22 U/d]; ETR 1.12 [95% CI 1.07 to 1.18]; *P* < .0001), whereas the mean bolus dose was statistically significantly lower with icodec (132 U/wk [∼19 U/d] vs 161 U/wk [∼23 U/d]; ETR 0.82; 95% CI, 0.74-0.90; *P* < .0001). Mean increases in body weight change from baseline to week 26 were similar between icodec (1.3 kg) and IDeg (1.0 kg; ETD 0.28 kg; 95% CI, −0.37 to 0.92; *P* = .41). Findings were similar at week 52.

The mean change in DTSQ total treatment satisfaction score from baseline to week 26 was statistically significantly lower for icodec (1.97) than for IDeg (3.06; ETD −1.09; 95% CI, −1.85 to −0.34; *P* = .0044) (120). Similar findings were observed at week 52. The authors suggest that this difference favoring IDeg may reflect this population of individuals with experience of once-daily basal insulins initially struggling with once-weekly insulin use.

Clearly, more studies in T1D are needed to complete the learning curve on how to better titrate icodec, ideally based on CGM profiles and not guided by the same titration regimens used for T2D that could well explain the differences in hypoglycemia seen in ONWARDS 6. Hopefully, the use of CGM for icodec adjustments may mitigate hypoglycemia risk in selected T1D populations.

#### Clinical pharmacology studies

One of the key preconceived concerns with once-weekly insulins is their potential for hypoglycemia compared to once-daily basal insulins. The 2 key questions that come up are 1) How long would an episode of hypoglycemia last? 2) Would the episode recur?

To investigate this risk, Pieber et al conducted a study comparing clinical, physiological, and counterregulatory hormone responses to double and triple doses of icodec with IGlar U100 in a 2-period crossover study in participants with T2D who were already on insulin ± oral glucose-lowering medication (124). Participants received either once-weekly icodec for 6 weeks or once-daily IGlar U100 for 11 days at equimolar total weekly doses based on the individual's run-in IGlar dose (mean 30 ± 14 units) and titrated to a target FPG of 80 to 130 mg/dL. Once at steady state, during each treatment period, a double dose and triple dose of icodec or IGlar U100 were administered followed by hypoglycemia induction at expected time of maximum glucose-lowering effect post dose (44 hours or 7 hours post dose for icodec or IGlar U100, respectively). Plasma glucose levels were initially maintained at euglycemia (100 mg/dL) by variable intravenous (IV) glucose/insulin and then allowed to decrease to a nadir of no less than 45 mg/dL with the discontinuation of the IV glucose infusion. Once nadir glucose was achieved, it was maintained for 15 minutes, following which the IV glucose was used to restore euglycemia. Validated hypoglycemia symptoms scores as well as cognitive tests were performed during hypoglycemia, and counterregulatory hormones were measured at nadir glucose. All patients also had real-time CGM performed through the treatment periods.

Clinically significant hypoglycemia (<54 mg/dL) occurred in a similar proportion of patients receiving overdoses of icodec or IGlar U100 (double dose: 40% vs 36%, respectively; OR 1.28; *P* = .63; triple dose: 53% vs 70%, respectively; OR 0.48; *P* = .14). Following a triple dose, the mean nadirs were 56 mg/dL for icodec vs 52 mg/dL for IGlar U100 (treatment ratio 1.07; *P* < .001). With each dose of icodec, the time it took to restore euglycemia was less than 30 minutes. The time to recovery with icodec vs IGlar U100 was similar following a triple dose but longer following a double dose. Counterregulatory hormone levels increased to a similar extent during hypoglycemia induction for both icodec and IGlar U100 with the exception of a slightly greater increase in adrenaline and cortisol in response to hypoglycemia following a triple dose of icodec. Symptoms related to hypoglycemia were also comparable between the icodec and IGlar U100 groups. Since both the hypoglycemia symptom scores and counterregulatory responses evoked by icodec was similar to IGlar U100, it appears likely that practices around hypoglycemia recognition and acute treatment that are currently in place for once-daily insulin analogues could also be applicable to once-weekly insulin treatment.

However, because of the long duration of action of icodec there is a risk of hypoglycemia recurrence. CGM data from this study showed time spent in hypoglycemia in the weeks following the double/triple doses was low even in those who had experienced clinically significant hypoglycemia (mean ± SD TBR [<54 mg/dL]: double dose 0.21 ± .45%; triple dose 0.56 ± 1.70%). The number of level 2 hypoglycemia events was also low from the end of the hypoglycemia induction experiments until 2 weeks after the icodec double dose (4 episodes in 3 participants) and until 1 week after the triple dose (6 episodes in 5 participants). Although these findings are reassuring, the study has a number of limitations: 1) patients at greater risk for hypoglycemia, those with renal failure, and individuals older than 72 years were excluded; 2) recovery from hypoglycemia was with a continuous infusion of IV glucose, not with traditional clinical measures such as administration of oral carbohydrate or glucagon; and 3) hypoglycemia recurrence risk was reduced by skipping the next scheduled dose of icodec (and IGlar U100) after hypoglycemia induction. Nonetheless, these data offer guidance on what to expect with inadvertent overdoses and also, skipping the next dose of the once-weekly insulin in the event of a significant hypoglycemic episode could reduce the risk of recurrence.

Several other studies in people at higher risk for hypoglycemia have been completed; people with renal impairment (NCT identifier NCT03723785) (125), or hepatic impairment (NCT identifier NCT04597697) (126). In the renal study, 58 participants with varying levels from renal function (normal renal function [n = 12], mild [n = 12], moderate [n = 12], and severe [n = 12] renal impairment, and end-stage renal disease [n = 10]) received a single SC icodec dose (1.5 U/kg) and were monitored for PK (125). The authors found that icodec exposure trended numerically slightly higher for patients with renal impairment compared to those with normal renal function (125). As discussed earlier, since this molecule is not renally excreted, these data do not suggest that doses of icodec will need to be modified based on its PK in renal failure but more so on the clinical characteristics of the patient. In patients with hepatic dysfunction, 25 participants with varying levels from hepatic function (normal hepatic function [n = 6], mild [n = 6], moderate [n = 6], and severe [n = 7] hepatic impairment) received a single SC icodec dose (1.5 U/kg) and were monitored for PK (126). The authors found that compared to participants with normal hepatic function, there was a slightly greater total icodec exposure with mild and moderate hepatic impairment, while no difference was observed for severe hepatic impairment (126). Again, the authors concluded that no specific dose adjustment of icodec is required in people with hepatic impairment.

### Insulin Efsitora

#### Phase 1 studies

The PK profile of efsitora was evaluated using single ascending doses (SADs) and multiple ascending doses (MADs) (108). In a 6-week SAD study conducted in healthy participants (n = 6 at each dose, 5 and 10 mg) and individuals with T2D (n = 6 at each dose; 10, 20 and 35 mg), efsitora administration resulted in glucose-lowering within 3 days of administration and led to a decrease in FPG that was dose-responsive and sustained for at least 5 days post dose (Fig. 11A). PK results showed that efsitora reached t_max_ at 4 days after dosing, with a mean half-life of approximately 17 days (range, 14.8-18.5 days) in individuals with T2D. Efsitora mean 7-point glucose profiles measured on days 4 and 43 (1 week after the final dose) remained constant and were similar to IGlar U100 (1 U/kg; n = 8). The rates and duration of hypoglycemic events with efsitora were similar to IGlar U100 (108).

In the MAD study, 33 individuals with T2D were randomly assigned to once-daily IGlar U100 or once-weekly efsitora. Based on the results of the SAD data and PK modeling, a loading-dose strategy was implemented to reduce the time to steady-state concentration. Individuals randomly assigned to efsitora received a one-time loading dose of 3 times their weekly dose. They then received a fixed dose (1, 2, 5, and 10 mg) once-weekly for the following 5 weeks. Individuals randomly assigned to IGlar U100 continued their usual dosing regimen throughout the study. The P/T ratio of efsitora concentrations over a 1-week period at steady state was determined to be 1.14. This indicates an approximately 14% increase in PK levels during the week from the time of injection. This P/T was calculated as the ratio of maximum concentration on day 4 after dosing to the concentration at 168 hours (7 days) post dose. Efsitora concentrations were flat across all dose levels (Fig. 11B). Unlike in the icodec PK study (86), it should be noted that with efsitora, a loading dose was used in this study to shorten time to steady state (Fig. 10B).

#### Phase 2 studies

Efsitora's phase 2 program included 3 treat-to-target studies: 1 study in patients with T2D previously treated with once-daily basal insulin (127), 1 in insulin-naive patients with T2D, and 1 in patients with T1D (128). In these phase 2 studies, efsitora was dosed in milligram increments from a reconstituted lyophilized powder since at the time of phase 2 studies the soluble insulin formulation was not yet available.

The first phase 2 study was conducted in patients with T2D already on basal insulin. The aim of this 32-week study was to assess not only efficacy but also frequency of titration as well as determine the optimal loading dose (127). A total of 399 participants were randomly assigned (1:1:1) to either of 2 once-weekly efsitora treatment groups with different fasting glucose targets and titration frequency or to a control group receiving once-daily IDeg. One efsitora group had a fasting glucose target less than or equal to 140 mg/dL with the insulin injected every week and titrated every 2 weeks, while the other had a fasting glucose target less than or equal to 120 mg/dL, again injected once a week but titrated every 4 weeks, that is, in the 2 efsitora groups, the dose could be changed every 2 or 4 weeks. Both efsitora treatment groups received a one-time loading dose ranging from 1.5 to 3 times their calculated weekly dose (127). The control group received IDeg U100 injected once a day and titrated every week to a fasting glucose target of 100 mg/dL or less. Participants used an unblinded Dexcom G6 for CGM. The primary objective of the study was to assess the change in HbA_1c_ from baseline.

Following 32 weeks of treatment, from a mean HbA_1c_ of 8.1%, there was a −0.6% reduction for both efsitora treatment groups and a −0.7% reduction for the IDeg group. Pooled analysis of the efsitora groups showed noninferiority in HbA_1c_ change vs IDeg. Level 1 hypoglycemia event rates were approximately 25% lower for the efsitora groups than the IDeg group. Level 2 hypoglycemia event rates were numerically lower for the efsitora groups compared to IDeg, but these differences did not reach statistical significance. However, fasting glucose levels were higher with both efsitora arms than with IDeg, which presumably could have ameliorated the hypoglycemia risk with efsitora (127). The data did demonstrate that irrespective of the higher fasting glucose levels in both efsitora arms, HbA_1c_ reduction was similar to IDeg, which had a lower fasting glucose. This could suggest better glucose control during the rest of the daytime with the longer-acting efsitora. These results were further supported by the study's CGM findings, in which during the 32-week treatment period, both efsitora groups and IDeg had similar TIR (70-180 mg/dL), TAR (>180 mg/dL), and TBR (≤70 mg/dL) over 24 hours. During the nighttime, participants in the efsitora group with a fasting glucose target of 140 mg/dL or less had significantly lower TBR (≤70 mg/dL) compared to IDeg probably driven by a higher glucose target (127). Additionally, at week 32 the duration of TBR was low and similar across the 7 days after injection of efsitora, showing that duration of hypoglycemia was not affected by the day post injection. However, these hypoglycemia data with efsitora will need to be confirmed in phase 3 trials, where more stringent fasting blood glucose targets of 80 to 120 mg/dL are being studied and what will actually be achieved in the trials.

These data on the discordance between fasting glucose and HbA_1c_ are similar to results from the icodec phase 3 studies discussed earlier, in which superior HbA_1c_ reductions were seen with icodec despite similar FPG levels as the comparator insulin, again suggesting that continuous weekly insulin exposure may be affecting glycemic parameters other than just fasting glucose.

People treated with efsitora had significantly smaller increases in body weight from baseline to week 32 (1.0 kg) compared with those treated with IDeg (2.0 kg) (127). Since exact unit dose conversion from mg to international units (IU) was not available for efsitora in this study, one cannot make an insulin dose comparison between efsitora and IDeg. The lower hypoglycemia rates, however, with efsitora could have contributed to the less gain in weight.

Additional phase 2 data comes from a 26-week, open-label study in insulin-naive patients with T2D, in which 278 patients were randomly assigned to 1:1 to efsitora once-weekly or IDeg once-daily (129). In the efsitora arm, weekly dose was determined based on median baseline fasting glucose and weight (129). The first dose was a one-time loading dose equal to 3 times the estimated weekly dose and ranged from 3 mg for someone with median fasting glucose of 140 mg/dL or less and body weight of 80 kg or less to 16.5 mg for someone with a median fasting glucose of more than 220 mg/dL and weight of 120.1 kg or more. From week 2, the participants received their calculated weekly dose, which was then titrated every week up to week 12, and then every 4 weeks thereafter to a fasting glucose goal of 80 to 100 mg/dL. IDeg was initiated at 10 units and titrated weekly to the same goal. Participants used a blinded Abbott Libre Pro for CGM during 14-day periods prior to weeks 0, 12, and 26. The primary end point was HbA_1c_ change from baseline to week 26.

From a baseline of 8.0%, efsitora (−1.20%) demonstrated noninferiority in HbA_1c_ reduction to IDeg (−1.26%; ETD .06 [90% CI −0.11-0.24]; *P* = .56) (129). The rates of level 1 and level 2 patient-reported hypoglycemia were similar between efsitora and IDeg (3.29 vs 2.77 and 0.22 vs 0.15 events/patient/year, respectively) with no severe hypoglycemia reported in either group. TIR (70-180 mg/dL) over a 24-hour period increased with both treatments for the 12- and 26-week assessments compared with baseline measures, with participants on both efsitora and IDeg having on average TIR 75% or greater over the 24-hour period by the end-of-study assessment. Efsitora demonstrated lower TBR (54- < 70 mg/dL) compared with IDeg (4.60% vs 7.06%; *P* < .1). There was no statistically significant difference in the body weight gain from baseline to week 26 between efsitora (2.9 kg) and IDeg (2.5 kg). Although no statistical analysis for change in insulin doses have been presented for this study, efsitora dose was numerically higher at study end, increasing from approximately 14 units/day at the beginning of the study to 51 units/day at week 26 compared to IDeg, which increased from approximately 10 units/day to 45 units/day at study end. The significance of this dose difference is not apparent at this time, and data from the ongoing phase 3 studies in similar populations will hopefully provide some answers.

In another phase 2 study in patients with T1D, the efficacy of efsitora vs IDeg was assessed in 265 patients over a 26-week treatment period (128). Participants in the efsitora arm received one dose of efsitora once-weekly with titration once-weekly for weeks 1 to 12 and every 4 weeks thereafter. Efsitora was initiated in a similar way as in the T2D insulin-naive population described earlier with a one-time loading dose. Since these patients were already on basal insulin, the one-time loading dose took into account the previous basal insulin dose, adjusted for fasting glucose, and then multiplied by a factor of 3. After this one-time dose, participants took their weekly dose based on their prior (prestudy dose) and titrated weekly to a fasting glucose target of 80 to 100 mg/dL. IDeg was self-administered once daily and titrated to the same target. Mealtime insulin adjustment was left at the discretion of the study investigators with guidance to follow standard of care. Participants used an unblinded Dexcom G6 for CGM. HbA_1c_ change from baseline to week 26 was the primary end point.

From a baseline of HbA_1c_ of 7.5%, efsitora demonstrated noninferiority to IDeg in HbA_1c_ change (0.04% and −0.13%, respectively; ETD 0.17%; 90% CI, 0.01-0.32; *P* = .07). Percentages of TIR (70-180 mg/dL) during the 24-hour period at week 26 were similar between treatment groups at week 26. The event rates for level 1 (efsitora: 207.6 and IDeg: 206.7 events/patient/year) and level 2 (efsitora: 40.7 and IDeg: 45.5 events/patient/year) hypoglycemia captured from CGM were similar for efsitora and IDeg. Similar durations of time in the hypoglycemic range were observed between efsitora and IDeg groups for both level 1 (28.4 vs 32.0 minutes; *P* = .371) and level 2 (7.46 vs 7.89 minutes; *P* = .82) hypoglycemia, with no prolonged or repeated hypoglycemia observed (128). People treated with efsitora had significantly smaller increases in body weight from baseline to week 26 (0.1 kg) compared with those treated with IDeg (0.6 kg; *P* = .028). There was no significant change in the basal insulin doses over the course of the study, and mealtime insulin doses were similar in both treatment groups, which might explain the minimal change in weight especially when coupled with small change in HbA_1c_.

It is noteworthy that in the phase 2 program efsitora was dosed in milligrams rather than in international units with the rationale that using phase 1 data to determine international units from insulin might not be the most accurate in all populations (130), and data from the phase 2 program would allow for a more appropriate calculation of the conversion to international units. As discussed earlier, in all 3 phase 2 studies, based on the PK needs to accelerate time to steady state, a one-time loading dose was administered (127-129).

Overall, in patients with T2D, in its phase 2 studies, efsitora achieved similar glycemic control to IDeg with no clinically significant differences in the rates of hypoglycemia. In the basal switch study, total and nocturnal level 1 hypoglycemia were significantly lower in efsitora titrated to a fasting glucose target of 140 mg/dL compared to IDeg, which had an fasting glucose target of less than 100 mg/dL, which may have contributed to this lower risk as discussed earlier (127). In this study, the duration of TBR with efsitora was similar irrespective of the day since the last injection (127). These hypoglycemia data with efsitora will need to be confirmed in phase3 trials, in which more stringent fasting blood glucose targets of 80 to 120 mg/dL are being studied. In both of the T2D studies, TIR metrics showed an improvement in TIR similar to IDeg and importantly periods of TBR especially at night were less than those seen with IDeg. These lower hypoglycemia findings with efsitora compared to icodec could be the result of differences in study design, glycemic control, and/or insulin titrations or perhaps influenced by efsitora's flat PK profile. Results from the ongoing Phase 3 studies will show if these initial observations continue to hold.

In patients with T1D, even with a tight fasting glucose target of less than 100 mg/dL, efsitora did not show a higher rate of hypoglycemia compared to IDeg. These findings were supported by TIR metrics, which did not show an increase in hypoglycemia, or its duration compared to IDeg. Retrospectively, when using all the data from the phase 2 program, the investigators indicated that efsitora was underdosed by approximately 30% in patients with T1D (128). This resulted in an initial period of hyperglycemia and led to a compensatory increase in the mealtime insulin to manage glycemia during the first couple of weeks. These observations highlight the importance of using a loading dose with the correct conversion factor and also suggest that there will be a learning curve for management of weekly basal dosing in patients with T1D.

#### Phase 3

Based on the phase 2 study results, efsitora has now initiated a phase 3 program, entitled QWINT (Once-Weekly [QW] Insulin Treatment), which consists of 5 clinical trials. All studies are currently ongoing. In the phase 3 studies, efsitora is formulated in solution and dosed in international units administered using prefilled insulin delivery devices.

Key design features of the QWINT trials are outlined in Table 2. QWINT 1 to 4 are treat-to-target studies in people with T2D that will assess efficacy and safety of efsitora compared to a once-daily comparator (IDeg or IGlar U100) in combination with noninsulin glucose-lowering medications. QWINT 1 compares a fixed dosing-escalation approach for once-weekly efsitora, with once-daily IGlar U100 as the comparator in insulin-naive patients. QWINT 2 is also studying an insulin-naive population, whereas QWINT 3 and 4 are in insulin-treated patients, the former in patients on basal insulin alone and the latter for those on basal-bolus therapy. QWINT 5 is studying people with T1D.

**Table 2.** Efsitora phase 3 trial design (QWINT 1-5)

	QWINT 1	QWINT 2	QWINT 3	QWINT 4	QWINT 5
Clinicaltrials.gov No.	NCT05662332	NCT05362058	NCT05275400	NCT05462756	NCT05463744
Population	T2D, insulin naive		T2D, previously insulin-treated		T1D
Key trial details					
**Primary objective**	**Noninferiority in HbA_1c_ change from baseline compared to once-daily comparator**				
Key secondary assessments	Superiority in HbA_1c_ change; rate and incidence of level 2 and 3 hypoglycemia; PRO measures	Superiority in HbA_1c_ change, TIR (70-180 mg/dL), and rate of nocturnal hypoglycemia; PRO measures	Superiority in HbA_1c_ change, TIR, and rate of nocturnal level 2 hypoglycemia	Superiority in HbA_1c_ and rate of nocturnal level 2 hypoglycemia	Superiority in HbA_1c_, TIR (70-180 mg/dL), and rate of nocturnal level 2 hypoglycemia
Randomized trial design	Open-label	Open-label	Open-label	Open-label	Open-label
No.	670*^a^*	912*^a^*	986	670*^a^*	692
Study start date	January 2023	June 2022	March 2022	August 2022	August 2022
Trial duration, wk	52	52	78	26	52
Main phase	52	52	26	26	26
Extension phase	—	—	52	—	26
Once-daily comparator	Glargine U100	Degludec	Degludec	Glargine U100	Degludec
Bolus insulin during study	—	—	—	Lispro	Lispro
Background medication	≥1 noninsulin glucose-lowering agent		0-3 noninsulin glucose-lowering agents	0-3 noninsulin glucose-lowering agents	—
Technology employed in study	—	CGM			
Key inclusion criteria					
Demographics	Adults aged ≥ 18 y				
HbA_1c_ at screening	7-10 (53.0-85.8)	7-10.5 (53.0-91.3)	6.5-10 (47.5-85.8)	7-10 (53.0-85.8)	7-10 (53.0-85.8)
BMI	—	≤45	≤45	≤45	≤35

**Abbreviations:** BMI, body mass index; CGM, continuous glucose monitoring; HbA_1c_, glycated hemoglobin A_1c_; MDI, multiple daily injections; PRO, patient-reported outcome; T1D, type 1 diabetes; T2D, type 2 diabetes; TIR, time in range.

^*a*^Estimated enrollment.

#### Clinical pharmacology

A 2-period, open-label clinical trial to evaluate the effect of efsitora compared to IGlar U100 in participants with T2D under conditions of increased hypoglycemic risk is reported in ClinicalTrials.gov as having completed data collection for primary outcome measure (NCT identifier NCT04957914), but no results have been posted or disclosed at the time of this writing.

## Potential Benefits and Concerns With Once-Weekly Basal Insulin

### Adherence and Persistence With Once-Daily Basal Insulins

Despite the availability of at least 4 different basal insulin analogues, there are still challenges both in the initiation of basal insulin (“clinical or insulin initiation inertia”) and, when initiated, achieving glycemic goals (“treatment or titration inertia”).

Multiple studies have shown that many patients and health care providers are reluctant to initiate insulin (initiation inertia) (131-135). A number of reasons for this clinical inertia have been proposed, with key factors including fear of needles and pain; concerns about side effects, especially hypoglycemia and weight gain; complexity of insulin dosing and glucose monitoring; and even potential effect on employment (135).

Even after insulin is initiated, only a minority of individuals reach recommended glycemic targets (6-8). Health care providers highlight multiple challenges with insulin titration (treatment inertia) with again concerns about side effects, especially hypoglycemia and weight gain, as well as a lack of resources to train patients, and concerns about patients’ potential for nonadherence (133, 136-138). Patients themselves also cite hypoglycemia and weight gain as concerns along with the perception that being on insulin means having a more severe disease. Complexity of dosing, and cost of insulin and the associated injection and monitoring supplies also play a substantial role in treatment inertia (131, 139-143). Combined, these barriers with insulin treatment result in not only the underachievement of glycemic targets, but also can entail long-term economic costs (144).

Multiple approaches have been tried to overcome initiation and treatment inertia with insulins, including diabetes self-management training, nurse- and pharmacist-led insulin management, increased psychological support, as well as advancements to simplify injection devices (131). Despite these interventions, however, challenges with once-daily basal insulin persist. Data from a large US database show that within the first year of initiation of basal insulin, almost half interrupted therapy in the first 3 months, with 15% of patients discontinuing insulin completely during these 3 months (145). Another study of electronic medical records of more than 40 000 individuals, this time from the United States and multiple European countries, showed that after insulin initiation there was an initial reduction of HbA_1c_ at 6 months after which HbA_1c_ plateaued, with less than a third of patients achieving an HbA_1c_ target of 7% or less at 24 months (146). What is, however, difficult to ascertain from these data is whether patients were actually taking the insulin as prescribed. In other words, assessing adherence to treatment in the real world is challenging and it may take more technological advances such as smart insulin pens to truly assess patient adherence.

Clearly, multiple barriers exist that affect success with once-daily insulin therapy and the availability of once-weekly basal insulins, and the associated significant reduction in the number of injections, may offer one promising option.

### Potential Advantages of Once-Weekly Insulins

#### Flexibility in time of administration

The stable and predictable PK profile of a once-weekly basal insulin has the potential to minimize patient burden and the micromanagement of insulin therapy that is currently required to maintain desirable glycemic control. These ultra-long-acting insulins would provide more flexibility in the timing of the dosing and may be more forgiving to dosing errors or skipped doses. Compared to once-daily basal insulins discussed earlier (Fig. 2B), once a once-weekly insulin reaches steady state it can be more forgiving and offer more flexibility than once-daily insulins, since skipping a dose may not result in an immediate or irremediable loss of efficacy given the long half-life of these drugs. Icodec dosing guidelines from their protocol offer guidance that for a missed dose it should be taken “as soon as possible” but if 3 days or fewer remain before the next dose, that week's dose should be skipped (116). There is precedence for this approach of skipping a dose with other weekly agents used in diabetes management, for example, dulaglutide and semaglutide (147, 148). As discussed earlier, IDeg, the once-daily insulin with the longest half-life, has also been studied for administration within an 8- to 40-hour window without showing loss of efficacy (59). With weekly insulins having a much longer half-life, similar principles of flexibility could apply.

Similarly, the flat PK profile of once-weekly insulins may also enable more consistent and perhaps less bolus dosing in patients on mealtime insulin since a steady basal insulin coverage over days, particularly during the night or between meals, is likely to reduce bolus needs. On the other hand, one could also argue that the increase in flexibility with dosing could lead to more complacency and worsening of glycemic control. Although real-world evidence would be the ultimate arbitrator for this concern, experience with long-acting GLP-1 receptor agonists (RAs) so far has shown that decreased frequency of injection does not decrease persistence to treatment (149).

When surveyed, both patients and health care providers indicate a preference for fewer injections both with insulin and GLP-1 RAs (137, 150-156). A reduction therefore in patient burden with a simplified, weekly dosing regimen, reducing the injection burden by 313 injections every year may lead to an improvement in adherence and persistence to insulin therapy. In addition, once at steady state, the frequency of testing blood glucose may also be reduced, lowering the treatment burden associated with insulin treatment.

In addition, digital health tools such as dosing guide apps may reduce barriers in insulin therapy and some such technologies available today have been shown to be associated with better glycemic control in people with T2D (157, 158). In the ONWARDS 5 study with icodec in insulin-naive T2D patients, real-world elements of once-weekly insulin using a dosing guide app were assessed. As discussed earlier, data indicate that this approach was successful, with superior HbA_1c_ reduction, higher insulin doses from continued titration, and similarly low rates of hypoglycemia with icodec used with a dosing app compared to standard of care using once-daily basal insulins. In addition, patient-reported outcomes from this study also indicate improved treatment satisfaction and compliance for icodec using the dosing app (115) Studies such as this provide useful insights about the possibilities of empowering patients to self-titrate their insulin.

#### Reduced glycemic variability

Fear of hypoglycemia and its potential consequences for patients, including cognitive dysfunction, can add to the stress of an insulin regimen (159, 160). If the flatter PK profile of once-weekly insulins could translate into a decrease in day-to-day (interday/between-day) glycemic variability, then there is a potential to reduce the emotional and physical burden of unpredictability with insulin therapy. Early data from an efsitora phase 2 study demonstrated lower within-day glycemic variability compared to IDeg. Between-day glycemic variability was also lower but only during the nighttime hours (127). However, these data should be interpreted with caution since fasting blood glucose targets were 20 to 40 mg/dL higher for efsitora compared to IDeg. These preliminary observations will need to be confirmed, and more data from the phase 3 trials are needed. The challenge with hypoglycemia assessment in these studies is that, at least in the patients with T2D when on basal insulin alone, overall hypoglycemia rates are extremely low making it hard to tease out differences between the once-weekly and once-daily insulins. When more CGM metrics are available from the phase 3 studies, there will be an opportunity to study differences not only in the within-day glycemic variability as is traditionally examined with once-daily insulins but also between-day variability, which may be a more important metric to assess with once-weekly insulins.

#### Patients that may benefit from once-weekly insulin

One could argue that any patient with T2D inadequately controlled on multiple glucose-lowering agents requiring basal insulin therapy, is a good candidate for a once-weekly insulin. Weekly insulins may well have greater acceptance simply based on the reduction in injection burden compared to once-daily insulins. Flexibility in dose timing may also be appealing to many. More specifically, patients with T2D who have difficulty with medication compliance may see significant benefits from the reduced injection burden, flexibility of dosing, and “forgiveness” when missing a dose.

A once-weekly basal insulin, particularly if combined with less aggressive glucose targets (161), may prove safer and provide a financial benefit for those patients that require a health care provider such as a caregiver to deliver and administer insulin since the total cost of insulin therapy includes these care visits in addition to the unit price of insulin. Such patients include older individuals and those in nursing homes and other extended care facilities. There is even the potential for these challenging populations to become more self-sufficient due to the stability of the glucose profiles over weeks instead of days because of the long duration of action of these insulins that can limit the need for multiple titrations. The same may be true for some people with T1D who have difficulty with medication compliance and who experience recurrent diabetic ketoacidosis (DKA) because of inconsistent insulin administration. In these patients, once-weekly insulins may provide benefit because of their stable and predictable profile considering that a common precipitating factor for DKA is insulin nonadherence, especially in teenagers (162). Furthermore, the long duration of action of these insulins could, in theory, restrain ketogenic hormone production.

Additionally, once-weekly insulins may enable clinicians to think differently about approaches to management of diabetes in ways that have not traditionally been apparent, which could lead to exploration of new treatment regimens. For example, could patients using insulin pumps who experience recurrent DKA potentially benefit from a low dose of once-weekly basal insulin in the background?

### Preconceived Concerns With Once-Weekly Insulins

#### Dose calculations

Despite the potential benefits of a once-weekly basal insulin regimen, there are several theoretical concerns with dosing. These insulins would represent a substantial transformation in current dosing regimens, which rely on once-daily basal insulin administration. Whereas the doses of once-daily basal insulins in use today are comparable between different insulin analogues, it will require effort from patients and health care providers alike to understand the new, weekly regimens. To initiate these insulins, weekly dose equivalents will need to be calculated, not only for insulin-naive patients, but also for those switching from a once-daily to a once-weekly treatment.

Several major differences in dosing between once-daily and once-weekly regimens are anticipated. First, since an entire week's basal insulin dose will need to be administered at one time, there will be a perception of risk that the dose is too large. These apparent large doses could in themselves add stress both for the patient and health care provider if it is not properly explained that these doses represent a standard daily dose that is now being added up for 7 days. Such explanations may help in alleviating concerns about the magnitude of these doses. For example, an insulin dose of 0.4 U/kg/day for a 70-kg individual will be approximately 196 units every week, which in daily equivalents is 28 units/day. Both health care providers and patients may, therefore, benefit from thinking in daily dose equivalents. Second, to shorten the time to reach a steady-state concentration, as discussed earlier, both icodec and efsitora have used a one-time loading (or starting) dose in clinical trials, which enables patients to achieve efficacious exposure more quickly compared to when no loading dose is given (see Figs. 8 and 10). This loading dose will likely be unique for each once-weekly basal insulin analogue based on differences in PK. Nonetheless, just the concept of a loading dose, although pharmacokinetically accurate and required, will no doubt cause angst both for patients and providers, highlighting the need for retraining on insulin-dosing principles. It is informative that these one-time loading doses have not induced any increased hypoglycemia risk over the initial weeks of the initiation of once-weekly insulins in studies so far.

#### Patients in whom once-weekly insulin may be challenging

In the views of the authors, because of the lack of endogenous insulin production and obtunded counterregulatory responses, patients with long-standing T1D represent a more challenging population for using once-weekly insulins. Given their slow onset of action, once-weekly insulins may not always be the best initial basal insulin in those with newly diagnosed T1D but may still be a good option since early T1D with some residual β-cell function may be easier to manage. However, as discussed earlier, increased hypoglycemia was seen in the icodec phase 3 T1D study compared to IDeg (ONWARDS 6) (120). In ONWARDS 6, rates of combined clinically significant or severe hypoglycemia were higher with icodec vs IDeg, although the authors note that rates were lower than those reported in previously published treat-to-target studies investigating IDeg in people with T1D. Additionally, the statistically significant treatment difference favoring IDeg vs icodec in DTSQ total treatment satisfaction score might suggest that the trial participants, who had experience with once-daily basal insulins, initially struggled with once-weekly insulin use. Although efsitora did not appear to increase hypoglycemia compared to IDeg in patients with T1D (128), this was in a phase 2 trial and one must wait for results from the ongoing phase 3 study (QWINT 5) before drawing any conclusions. Real-world experience on how to best titrate both the once-weekly basal and mealtime insulins in people with T1D will also help in determining the best way to dose in this population. Overall, the currently available data with once-weekly insulins in T1D in adults should be regarded only as preliminary and more data especially with CGM based metrics might be required to learn how to minimize hypoglycemia risk with these insulins in people with T1D. In addition, if an indication is sought for a pediatric population with T1D, a careful assessment of data specific to this population would be needed.

Similarly, these insulins are not appropriate to initiate in patients hospitalized with acute illnesses, since they can take weeks to achieve glycemic control, and basal insulin with a more rapid onset of action and shorter half-life is preferred in this circumstance.

## Clinical Implications

### Implications of Dosing Differences Compared to Once-Daily Basal Insulins

#### Switching between once-weekly and once-daily basal insulins

The ability to switch from a once-daily basal insulin to a once-weekly insulin and vice versa has been investigated, in part, in the clinical trials as patients initiated and terminated the study drugs. In phase 2 studies that have been reported so far, the transition to a once-weekly basal insulin at the start of the reported trials and the transition back to a once-daily basal insulin did not appear to result in adverse consequences. In addition, as previously discussed, TIR data from 2 icodec phase 3 studies at the time of the switch from once-daily basal insulin to icodec showed that such switches did not lead to a loss of glycemic control or more hypoglycemia when a loading dose was administered (122, 123). In the ONWARDS 1 study in insulin-naive patients, at study end, according to the study protocol, the first dose of the once-daily insulin post trial was administered after a 2-week gap from the last icodec dose accompanied by recommendations for more frequent monitoring of glucose (116). We should learn more as glucose data at the time of switch back to once-daily insulins from the completed studies become available. These data will be particularly valuable if there is CGM information overlapping the time of the switch and a few weeks beyond.

#### Monitoring glucose responses with once-weekly basal insulins

Given the long duration of action of these insulins and with increasing access to CGM technology, monitoring the response to therapy with once-weekly insulins may be facilitated by TIR measures (163). As discussed earlier, data from the icodec phase 3 studies show a lack of concordance between FBG reduction and HbA_1c_; for similar FBG reductions to comparator daily insulin, icodec achieved a superior HbA_1c_ change (115-119). In a phase 2 study, efsitora also achieved a similar HbA_1c_ reduction with lower hypoglycemia compared to IDeg when FBG targets were set to be 20 to 40 mg/dL higher than IDeg (127). These findings generate 2 clinical questions: 1) Is FBG the ideal way to monitor response to therapy with weekly insulins? 2) Are FBG targets that are standardized for once-daily basal insulins appropriate for weekly insulins? Although clinical trials are still using fasting glucose and treat-to-target methodologies with narrow fasting glucose targets as mandated by regulators, in clinical practice, even though FBG may still be the parameter to titrate the dose of weekly insulin, the actual response to therapy might be better assessed with CGM since it would provide more details on glycemic trends than a unitary FBG measure. Second, widening of the FBG targets beyond the treat-to-target goals of 80 to 130 mg/dL used in regulatory studies may be an approach that could be considered with once-weekly insulins even in the absence of CGM. These widened targets, such as those used in one arm of the efsitora phase 2 study (FBG target ≤140 mg/dL) (127), could potentially reduce the risk of hypoglycemia compared to once-daily insulins. Although such a change in target range may compromise achieving stringent HbA_1c_ goals, these targets may be appropriate especially in some high-risk populations such as older individuals or people with advanced cardiovascular risk or CKD. Additional analysis of CGM data from ongoing and completed clinical trials with these molecules could help inform some of these clinical implications.

### Hypoglycemia Evaluation

To assess hypoglycemia, one will still need to use traditional monitoring measures in clinical trials to determine nocturnal, total, and severe hypoglycemia rates to provide reassurance to clinicians and patients as they transition to once-weekly insulins.

In phase 2 and 3 trials in patients with T2D, self-monitored blood glucose has been used for titration and hypoglycemia evaluation; study design protocols are clear and easily adoptable for clinical practice (88, 116). In clinical practice, during the titration phase when these insulins are initiated, at a minimum, more frequent monitoring will be needed not only to gauge glycemic response but also for hypoglycemia detection. One can argue that CGM would be useful in this regard since it will provide data over not only the course of 1 day but the whole week, allowing for monitoring for recovery as well as recurrence of hypoglycemia (163). In addition, CGM glycemic trends may allow for proactive dose changes to try to preempt hypoglycemia (or hyperglycemia) since any dose change with a weekly insulin might not manifest itself for a few weeks, unlike with a daily basal insulin when the change is manifest within the next 24 hours. In the ongoing and completed studies with icodec and efsitora in T2D, CGM was used, for the most part, in a blinded fashion to collect data and not for therapeutic intervention. More recently, icodec has initiated a study in adults with T2D where a flash CGM is being used for titration of the insulin (NCT identifier NCT05823948). Once these data are available, they may help further inform the utility of CGM for clinical practice with once-weekly insulins. The major downside of this approach, however, is that not every patient will have access to CGM technology.

### Management in Common Clinical Scenarios

As the half-lives of once-daily basal insulins have been prolonged, health care providers have learned both through clinical practice and real-world and clinical pharmacology studies how to manage dosing in common clinical scenarios such as hypoglycemia, hospitalization, fasting (due to medical procedures, religious reasons, weight management) as well as exercise. Some of these learnings may be extrapolated to once-weekly insulins.

#### Hypoglycemia management with once-weekly insulins

A number of factors may affect recovery from hypoglycemia or lead to prolonged or recurrent hypoglycemia in people with diabetes. Prolonged hypoglycemia can result from (a) failure to generate an appropriate glucagon and other counterregulatory hormone response, which is mainly applicable in T1D but can also occur in long-standing T2D; (b) failure of insulin to dissipate; and (c) failure to recognize the precipitating factors responsible for the episode and take corrective action to prevent recurrence. Given the long half-life of once-weekly insulins, it is important to consider not only how to manage an acute episode but how to best monitor for recurrence or persistence of hypoglycemia were an episode to occur.

As discussed earlier, at least with the T2D population, icodec has a similar counterregulatory hormone response and recovery compared to IGlar U100 during an acute episode of hypoglycemia (124). This is reassuring and suggests that from the perspective of management of an acute episode, the fundamental principles should be no different to those with once-daily basal insulins: administer calculated amounts of carbohydrates, monitor response, and repeat as necessary. These principles appear to be working in the phase 2 and phase 3 programs with these once-weekly insulins as there was no evidence presented of a delay or resistance to recover from level 2 hypoglycemia or even from the small number of severe hypoglycemic episodes.

Post hoc analysis of icodec CGM data showed that irrespective of the titration algorithms used or the presence of a loading dose, the duration of a hypoglycemic episode was similar with both icodec and IGlar U100 (114, 123). With efsitora, the duration of time spent in hypoglycemia (both level 1 and 2) as measured by CGM was similar across all 7 days post injection in a phase 2 basal switch study (127). It is, however, important to note that randomized clinical trials are generally conducted in low-risk populations and risk of hypoglycemia may be higher in the real world and that recurrence may not necessarily be directly due to the insulin itself but rather to other underlying medical conditions, errors with dosing, dietary noncompliance, or not following hypoglycemia management instructions. Until more data from the clinical trials becomes available and clinical experience accrues with these insulins, at the very least, more frequent monitoring over a few days following a hypoglycemic event would be prudent. Such monitoring might be especially important during the nighttime hours when endogenous glucose production is the only source of carbohydrates. In addition, since the once-weekly insulin would take longer to dissipate compared to once-daily insulins, one could argue that if frequent level 1 hypoglycemia occurs (which is considered an alert level) this could be a trigger to widen the glucose targets and/or initiate a proactive reduction in the dose of the once-weekly insulin, bearing in mind that the effect of the reduced dose might not manifest immediately. On the other hand, a level 2 episode or even frequent level 1 episodes might bring up the consideration of not only reducing the next dose but perhaps even skipping a dose entirely as was done in the icodec clinical pharmacology study when hypoglycemia was precipitated in a controlled setting (124). These cautions would be particularly important in people with very tightly controlled HbA_1c_, older individuals, those who are eating less or who are losing weight, and patients with renal dysfunction (CKD).

#### Management during hospitalization, fasting, and exercise

During hospitalization and for surgeries, patients and health care providers would need to consider the implications of being on an ultra-long-acting basal insulin. These scenarios were of concern when ultralente was first introduced (18), and then again during the development of IDeg, which is currently the once-daily basal insulin with the longest half-life (164). Although not realized in clinical practice, these concerns are real and need to be considered with every new long-acting basal insulin including once-weekly insulins. To the best of our knowledge, there have been no specific reports on study participants who have been admitted to hospital while using these once-weekly insulins in the phase 2 or 3 studies. Protocols from the phase 3 program on how these common clinical situations were managed in the studies should offer some clues, but dedicated hospital studies and real-world experience will truly inform clinical practice. As discussed earlier, research is currently evaluating the effects of efsitora as compared to IGlar on frequency and severity of hypoglycemia in situations where such risk increases (exercise and fasting) (NCT identifier NCT04957914), but no data are available at the time of this writing.

In the opinion of the authors, patients in the hospital for protracted illness and those requiring a steady source of enteral or parenteral nutrition might theoretically benefit from continuing once-weekly basal insulin if they were already on it and were at steady state prior to being admitted since they would have a steady source of insulin to meet basal metabolic needs. Similarly, short overnight or less than 24-hour hospital stays might not require any change in once-weekly insulin that was already being administered as an outpatient when the patient is on stable doses. However, these patients will still require close glucose monitoring to detect hypoglycemia or to supplement with a rapid-acting insulin in case of unwanted hyperglycemia.

**Figure 12.**
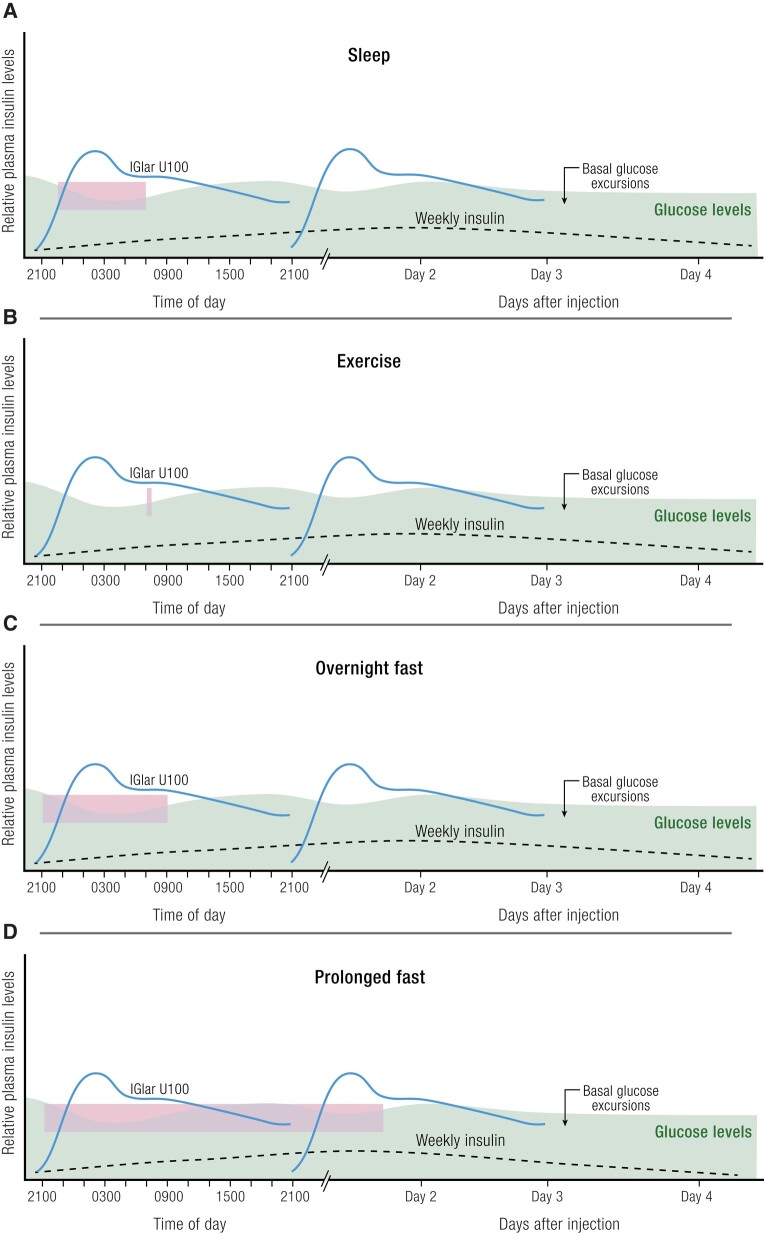
Schematic representation of the potential effect of sleep, exercise, and overnight and extended fasting (red boxes) on icodec and efsitora dosed once-weekly compared to IGlar U100 dosed at 2100 hours daily. A, Following dosing at 2100 hours, peak IGlar U100 concentration would be expected in the early morning hours, whereas with weekly icodec or efsitora, there would be minimal difference in insulin exposure. B, A 30-minute period of exercise at around 0700 hours is depicted. In this example, exercise would occur either at the peak action or shortly thereafter of an IGlar U100 dose administered at 2100 hours. With icodec or efsitora given the constant exposure of insulin concentrations without a peak, the effect of the exercise on glucose levels would be more predictable. C, With overnight fasting, IGlar U100 could have a peak in the early morning hours that could increase the risk of hypoglycemia and a dose reduction of the IGlar on the night of the fast may be prudent. With icodec and efsitora no change in dose will be needed. D, With a prolonged fast, for example, following major abdominal surgery or similar event, where the person is dependent on endogenous glucose or an exogenous glucose source, based on target range of glucose for the patient, with weekly insulins no intervention may be acceptable. With IGlar, multiple dose adjustments may be required.

Using PK schematics, one can create scenarios comparing once-weekly insulins and the most commonly used basal insulin IGlar as depicted in Fig. 12 that might help clinicians understand and develop protocols for management with once-weekly insulins in common clinical situations such a fasting and exercise.

### Implications of Once-Weekly Insulins as Combination Therapy With Glucagon-like Peptide-1 Receptor Agonists

Since approval of the first incretin for treatment of T2D in 2005, GLP-1 RAs are now widely and successfully used (165). This increased use is driven not only by the availability of newer and more potent GLP-1 RAs and glucose-dependent insulinotropic polypeptide (GIP)/GLP-1 RA formulations (tirzepatide), but also by their cardiovascular benefits and reduced frequency of injections compared to the daily injection of the first-generation compounds. These benefits have resulted in changes in guidelines to recommend GLP-1RAs as first-line agents (17). Guidelines also recommend that if insulin is to be used, it should be used in combination with GLP-RAs both for greater efficacy and as well as durability of its effects (17). These recommendations are based on data showing that GLP-1RAs when combined with basal insulin either as separate agents or in fixed-ratio combinations offered both improved HbA_1c_ efficacy and favorable effects on weight and hypoglycemia risk (166).

However, considerable delay in intensification of treatment with addition of insulin has been reported in patients with T2D despite being inadequately controlled with GLP-1 RAs (167). Given the similar frequency of injections, once-weekly basal insulins may facilitate a simplified integration with once-weekly incretin therapies. Both drugs could be administered as separate injections or as one combined fixed-dose preparation. One such fixed-dose combination of icodec and GLP-1 RA, semaglutide (IcoSema), is currently in 3 phase 3 studies (COMBINE 1 (NCT05352815), COMBINE 2 (NCT05259033), and COMBINE 3 (NCT05013229) to evaluate the efficacy and safety of this combination therapy approach.

## Future Considerations

With efsitora in late-phase development and icodec already submitted for regulatory approval, it is reasonable to think through future considerations that could come into play were these insulins to be approved.

For patients with T2D, ADA/European Association for the Study of Diabetes guidelines recommend the use of basal insulins with the lowest propensity to cause hypoglycemia (17, 168). Although CGM metrics for hypoglycemia (TBR) are increasingly used in clinical practice and collected in most phase 3 once-weekly insulin studies, they are currently not accepted by regulators or guidelines as a means to compare hypoglycemia rates. However, recent draft guidance from the US Food and Drug Administration (FDA), if approved, may affect these limitations (169).

The FDA and other regulatory agencies recommend a treat-to-target approach when studying insulin in a clinical trial (170). The critical step in this approach is to set a fixed (and narrow) fasting glucose range, most commonly 80 to 120 or 130 mg/dL. The test insulin and the comparator are both then titrated to reach this glycemic target so other outcome measures such as hypoglycemia or weight gain can be properly evaluated. The reason for this approach is to set a level playing field that allows for the comparison of secondary effects when both the test insulin and comparator standard-of-care insulin have the same degree of glycemic control. This methodology has worked well so far when one was comparing a once-daily insulin with another once-daily insulin. However, comparing an insulin with a half-life of approximately 8 days (icodec) or approximately 17 days (efsitora) with either IGlar U100 (t_1/2_ 12-15 hours) or IDeg (t_1/2_ ∼25 hours) may not create a level playing field for testing secondary outcome measures given such different pharmacokinetics. The longer duration of action could give a once-weekly insulin an advantage in efficacy since it would be available for glucose metabolism even when the comparator once-daily insulin has reached a PK nadir, a nadir it will hit almost every day or in some cases before the day is done. This advantage for once-weekly insulins manifested itself in the results from the icodec phase 3 program, which demonstrated superior HbA_1c_ reduction compared to both IGlar U100 and IDeg at primary end point in 4 of the 5 studies in T2D patients (115-119). Moreover, as discussed earlier, despite similar FBG reduction to the comparator once-daily insulin, icodec was able to demonstrate better HbA_1c_. The improved HbA_1c_ must therefore be the result of the effect of the weekly insulin at other times of the day, an effect that may be measurable with CGM. At this time, however, there is no regulatory path to either study insulins using CGM metrics or to promote the data from CGM metrics.

This landscape, however, may be changing. Recently, the FDA has released draft guidance addressing 2 issues: 1) the use of CGM in clinical trials and 2) hypoglycemia assessment as an efficacy end point in clinical trials. According to this guidance, the use of CGM to assess TIR may be acceptable but only as an additional efficacy end point. The primary end point will still need to be HbA_1c_. The FDA also acknowledges that CGM may carry advantages over self-monitored blood glucose in the assessment of hypoglycemia given its ability to detect hypoglycemic episodes that could be missed by self-monitored blood glucose testing. However, to use hypoglycemia as a safety/efficacy end point, the FDA considers a reduction in level 3 hypoglycemia to be the preferred measure for a claim of safety/efficacy, provided both the test and control group achieved equipoise or similar HbA_1c_ reduction. In situations where hypoglycemia risk was expected to be low, a composite of level 2 and 3 may be acceptable. In addition, any CGM technology that is used needs to have been appropriately validated and assessed by the FDA. A full discussion of the guidance is beyond the scope of this review, and the reader is directed to the FDA draft for details (169).

The challenge, however, still remains that even with the new guidance, there does not seem to be a clear path on how to compare the effect of once-weekly insulins with those from once-daily insulins to have clinically relevant interpretations of both efficacy and safety. Standardized CGM metrics of TIR, TBR, and within-day glycemic variability have been developed for once-daily and mealtime insulins and pump therapy. New metrics that take into account the extended PK profiles of weekly insulins may need to be developed to accurately assess glycemic changes with these molecules. There are no clear and easy answers at this time but more deliberations among clinicians, researchers, and regulators are needed.

There is ongoing concern about the cost of insulin, particularly in the United States, the reasons for which have been extensively covered elsewhere (171, 172). If approved, once-weekly insulins can potentially offer substantial advantages, especially in delivering insulin to the frail in the community where assistance may be required for dosing and so any reduction in the frequency of these can be particularly beneficial (173). In addition to the reduced injection burden compared to once-daily insulins, once at steady state, the frequency of self-glucose testing may also be reduced. With efsitora, for example, titrating every 2 or 4 weeks produced similar reductions in HbA_1c_ compared to titrating every week with once-daily IDeg (127). Having an insulin with a very long half-life many also allow glycemic control to be maintained in the event of an inadvertent missing of a dose, which could be a considerable advantage to those people who skip doses.

## Conclusion

Since its discovery more than 100 years ago, insulin therapy has advanced significantly with safer and more efficacious iterations of the hormone in a quest to mimic endogenous action. Once-weekly insulins are the latest advance with potential to provide a significant transformation in basal insulin therapy. Two molecules, icodec and efsitora, have reached advanced stages of clinical development with the possibility of reaching patients within the next few years.

Both molecules create a circulating reservoir of insulin with the sustained release of active insulin that can engage the IR. Icodec achieves this by conjugating with HSA while efsitora is composed of a novel single-chain variant of insulin fused to a human IgG2 Fc domain. Both molecules have large hydrodynamic sizes and have reduced IR affinity compared to native insulin, limiting internalization and IR-mediated clearance. These molecular properties attenuate transport across capillary endothelium, limit activity, and prolong time-action and thus facilitate once-weekly administration. The main differences between the 2 molecules lie in their half-lives, which are approximately 8 days for icodec and approximately 17 days for efsitora. These differences likely translate into a more rapid time to steady state for icodec but a flatter PK profile for efsitora.

From the data we have so far, both once-weekly insulins appear as efficacious as once-daily basal insulins. Overall frequency of hypoglycemia is low, and level 2 and 3 hypoglycemia rates so far are not clinically significantly different from once-daily basal insulins in people with T2D. In people with T1D, however, there is reason for caution until additional data are available but overall we are just at the beginning of the learning curve how to use once-weekly insulins in these patients. More research, including data from CGM metrics on both hypoglycemia and hyperglycemia from both phase 3 programs, will be informative but to fully establish the hypoglycemia and safety profile of these insulins, longer evaluation in clinical practice will be required.

These insulins, however, do offer the enticing possibility of a major change in how we administer basal insulin. While the uniqueness of their dosing compared to daily basal insulins will require substantial investment in time and effort on the part of the health care community, these molecules have the potential to become “game changers” to improve acceptance, adherence, and persistence on insulin therapy because of the significant reduction in injection burden. If approved for use, real-world experience with these weekly insulins will be the ultimate arbitrator of their success.

## Abbreviations

ADA: American Diabetes Association
CGM: continuous glucose monitoring
CKD: chronic kidney disease
DKA: diabetic ketoacidosis
DTSQ: Diabetes Treatment Satisfaction Questionnaire
ERR: estimated rate ratio
ETD: estimated treatment difference
ETR: estimated treatment ratio
FcRn: neonatal Fc receptor
FDA: US Food and Drug Administration
FPG: fasting plasma glucose
GLP-1: glucagon-like peptide-1
HbA_1c_: glycated hemoglobin A_1c_
HGP: hepatic glucose production
HSA: human serum albumin
IDet: insulin detemir
IGF-1: insulin-like growth factor-1
IGF-1R: insulin-like growth factor-1 receptor
IgG: immunoglobulin G
IGlar: insulin glargine
IR: insulin receptor
IV: intravenous
MAD: multiple ascending dose
MAPK: mitogen-activated protein kinase
NPH: neutral protamine Hagedorn
PD: pharmacodynamic
PK: pharmacokinetic
P/T: peak-to-trough
PYE: patient-year of exposure
QWINT: Once-Weekly (QW) Insulin Treatment
RA: receptor agonist
SAD: single ascending dose
SC: subcutaneous
SCI: single-chain variant of insulin
t_1/2_: half-life
T1D: type 1 diabetes
T2D: type 2 diabetes
TAR: time above range
TBR: time below range
TIR: time in range
