# Correction to: “The Basis for Weekly Insulin Therapy: Evolving Evidence With Insulin Icodec and Insulin Efsitora Alfa”

**DOI:** 10.1210/endrev/bnae012

**Published:** 2024-03-22

In the above-named article by Rosenstock J, Juneja R, Beals JM, Moyers JS, Ilag L, and McCrimmon RJ (*Endocrine Reviews* 2024; doi: 10.1210/endrev/bnad037), there was an error in Figure 10.

In the above-mentioned article, the Figure 10A caption read: “Schematic depiction of the distribution of efsitora in the different biological compartments over time from initiation of once-weekly dosing (injection 1) through injection 8, showing the gradual movement of insulin icodec from the subcutis through the blood to the intercellular space where build-up occurs.” In the corrected articled, “insulin icodec” has been changed to “insulin efsitora” and reads as: “Schematic depiction of the distribution of efsitora in the different biological compartments over time from initiation of once-weekly dosing (injection 1) through injection 8, showing the gradual movement of insulin efsitora from the subcutis through the blood to the intercellular space where build-up occurs.”

The article has been corrected online.

doi: 10.1210/endrev/bnad037

